# Anthracnose drives assembly of phyllosphere epiphytic bacterial communities to increase disease resistance

**DOI:** 10.1038/s41522-026-00991-z

**Published:** 2026-05-15

**Authors:** Rongchun Zheng, Yingde Li, Panpan Shang, Youlei Shen, Zhibiao Nan, Tingyu Duan

**Affiliations:** 1 State Key Laboratory of Herbage Improvement and Grassland Agro-ecosystems, Lanzhou University, Lanzhou, China; 2 Key Laboratory of Grassland Livestock Industry Innovation, Ministry of Agriculture and Rural Affairs, Lanzhou, China; 3 Engineering Research Center of Grassland Industry, Ministry of Education, Gansu Tech Innovation Centre of Western China Grassland Industry, Lanzhou, China; 4 College of Pastoral Agriculture Science and Technology, Lanzhou University, Lanzhou, China

**Keywords:** Ecology, Ecology, Microbiology, Plant sciences

## Abstract

The phyllosphere microbiome plays crucial roles in plant health, but evidence of ‘cry for help’ strategy in the face of pathogen attack in the phyllosphere remains limited, particularly for the microbiomes of distinct leaf ecological niches. We investigated whether foliar pathogen anthracnose (*Colletotrichum lentis*) influenced the assembly and functions of microbiomes in epiphytic and endophytic niches of the phyllosphere of common vetch (*Vicia sativa*) leaves. We also evaluated synthetic microbial communities (SynComs), including representatives of disease-associated strains, for pathogen protection. Anthracnose mediated the deterministic assembly process of epiphytic bacterial and endophytic fungal communities, and increased the complexity of bacterial co-occurrence networks. Iron competition and antifungal genes were also enriched in the epiphytic bacteria, which produce siderophores and degrade fungal cell walls to counteract pathogens. SynComs of beneficial epiphytic bacteria partially protect hosts by regulating bacterial interactions and inducing host immune responses. These findings suggest that disease drives the deterministic assembly of distinct phyllosphere microbiomes, their diversity and their function. Moreover, SynComs from the epiphytic niche can confer host plant disease resistance.

## Introduction

Phyllosphere microorganisms are diverse and include bacteria, fungi, viruses, algae, nematodes and protozoa^1^. Phyllosphere microorganisms can inhabit the leaf surface as epiphytes or within the leaf as endophytes^2^. The phyllosphere is one of the largest microbial habitats, with high species diversity and pivotal roles in plant health and ecosystem functioning^3,4^. It is exposed to a wide range of biotic factors, including interactions with pollinators and insect herbivores and abiotic factors, including ultraviolet radiation, rainfall and heat, as well as anthropogenic pressures such as agricultural practices^4^. These diverse factors contribute to the dynamic and heterogeneous nature of the phyllosphere^5^, as well as the microbial communities inhabiting this niche. Understanding the implications of environmental changes for phyllosphere microorganisms is critical for sustaining plant health.

The plant microbiome can increase the coding and metabolic capabilities of host genes, supporting essential biological functions such as nutrient absorption, immune regulation and resilience to biotic stressors^6^. Although the rhizosphere microbiome has been the historical focus of research, the phyllosphere microbiome has an equally critical role in preserving plant health. The adverse effects of plant pathogens, especially foliar pathogens, on leaves have posed a persistent challenge to global food security for centuries^7,8^. Limited evidence suggests that, similar to rhizosphere microbes, plants infected by foliar pathogens can use a ‘cry for help’ strategy to attract beneficial microbes from the surroundings and increase their beneficial functions^9,10^. Nevertheless, the mechanisms governing the composition and assembly of phyllosphere microbiota and their roles in influencing plant health remain unresolved.

The phyllosphere microbiome can confer disease resistance by regulating microbial interspecies interactions, host metabolism and immunity^7,11^. Pathogenic and nonpathogenic microorganisms directly compete for space and nutrients within the phyllosphere. Additionally, phyllosphere microorganisms produce diverse secondary metabolites with pivotal ecological roles in both promoting and inhibiting microbial activities. For instance, 2,4-di-tert-butylphenol, synthesised by the phylloplane fungus *Aspergillus cvjetkovicii*, disrupts the intracellular reactive oxygen species levels of pathogenic fungi such as *Rhizoctonia solani* and *Fusarium fujikuroi*, consequently repressing their expression of AMT1 genes, which can attenuate disease-causing abilities of the pathogenic fungi^12^. Phyllosphere microorganisms can also regulate host gene expression and aid in defence against pathogen-induced stress. They can modify plant volatile organic compound emissions by eliciting plant defence responses or perturbing normal metabolism^13^. Moreover, nonpathogenic microbiota can also mediate host immunity, assisting the plant in suppressing invading pathogens^14^.

Epiphytic and endophytic compartments of the phyllosphere represent separate ecological niches with highly diverse microenvironments, thereby supporting communities of epiphytes and endophytes with contrasting composition and diversity^15,16^. Leaf epiphytic surfaces have higher microbial diversity and abundance than those of the leaf endosphere compartment^17^. Epiphytic microorganisms, exposed to the external environment, depend on nutrients deposited on or secreted by the leaves, whereas endophytic microorganisms inhabit the internal environment and obtain nutrients from host tissues^18^. Environmental factors, as well as host genotypes, determine the abundance and diversity of phyllosphere microbiota^5,19^. Notably, the responses of epiphytes and endophytes diverge in the presence of leaf-infecting pathogens. For example, the diversity and functional changes in epiphytic bacteria exceeded those in endophytic bacteria following melanose (*Diaporthe citri*) infection of citrus leaves^9^. Furthermore, microbiomes associated with plant aerial surfaces induce metabolic disease resistance in rice, protecting it against rice false smut (*Ustilaginoidea virens*)^13^. Nevertheless, although phylogenetic diversity is greater in the epiphytic microbiome, it does not possess the same level of direct inhibitory activity against plant pathogens as the endophytic microbiome^20^.

Most studies on phyllosphere microbial disease antagonism traditionally focus on the leaf as a unified entity, with little exploration of distinct phyllosphere ecological niches. In common vetch (*Vicia sativa*), the roles of disease in shaping the phyllosphere community have not been investigated. Therefore, we compared the phyllosphere microbiomes of healthy vetch leaves and those infected with anthracnose (*Colletotrichum lentis*) using a combination amplicon sequencing, metagenomic sequencing and culture-dependent methods to (i) analyse alterations in the phyllosphere microbial communities across distinct ecological niches of healthy and diseased leaves; (ii) explore the potential functional changes in the phyllosphere microbiota in supporting plant health under pathogen attack; and (iii) assess the efficacy of disease inhibition and the underlying mechanisms of synthetic communities (SynComs) composed of core phyllosphere bacteria induced by anthracnose.

## Results

### Alteration of phyllosphere microbiota diversity, structure and assembly

**Fig. 1:**
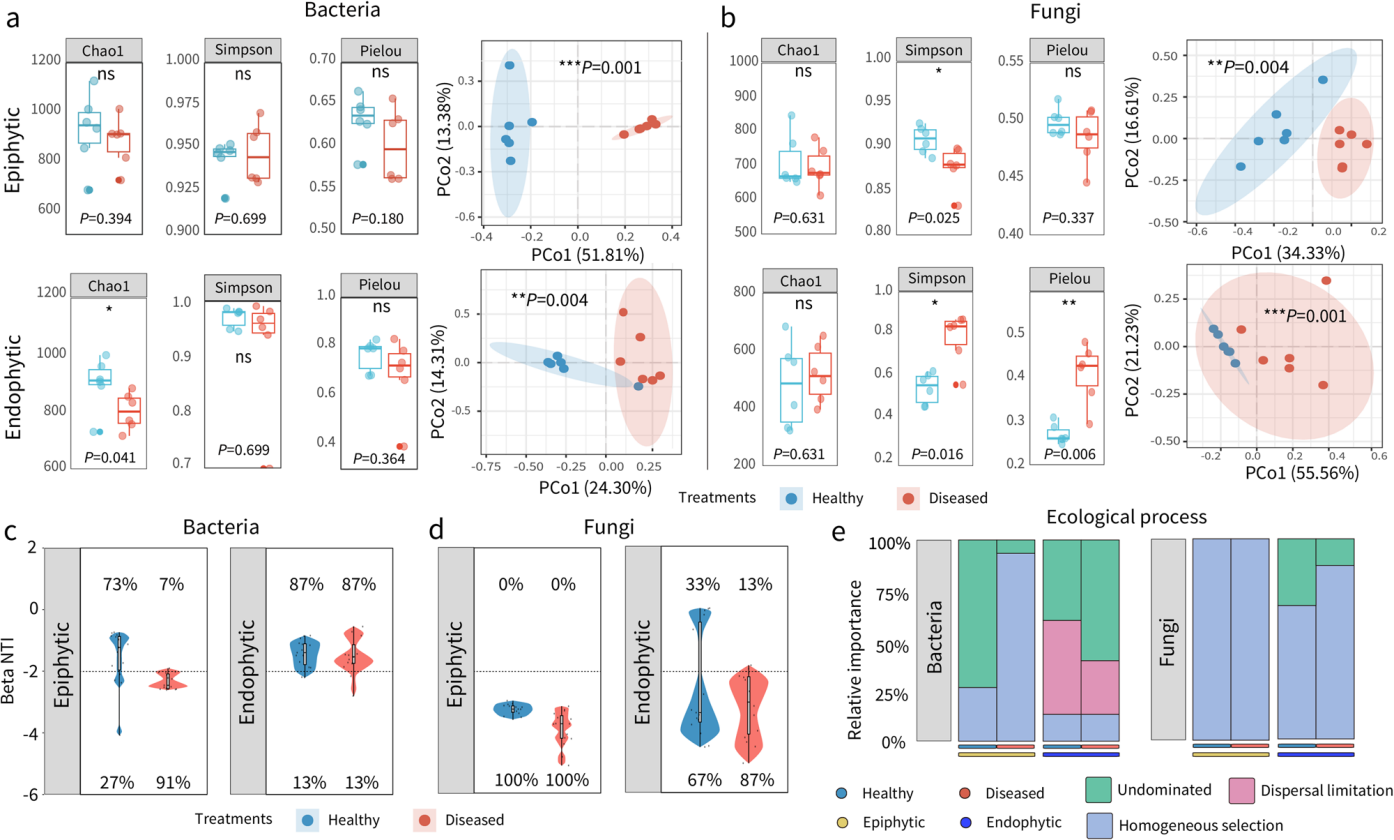
Disease-induced alteration of phyllosphere microbial community structure and diversity. **a** Alpha and beta diversity of epiphytic and endophytic bacterial communities in healthy and diseased leaves. **b** Alpha and beta diversity of epiphytic and endophytic fungal communities in healthy and diseased leaves. Data are shown as boxplots, where the centre line = median; box limits = first quartile (Q1, lower) and third quartile (Q3, upper); whiskers = min/max values within 1.5× interquartile range (IQR); and points = outliers. The *P*-values for alpha diversity indices indicate a significant difference between healthy and diseased leaves at *P* < 0.05 determined via Wilcoxon test. Principal coordinate analysis (PCoA) was conducted based on Bray–Curtis distances to characterise beta diversity, with PERMANOVA performed to test for significant differences between healthy and diseased leaves. *P < 0.05, **P < 0.01, ***P < 0.001, ns, no significance. **c** Relative contributions of deterministic and stochastic processes to the assembly of phyllosphere epiphytic and endophytic bacterial communities in healthy and diseased leaves based on β-nearest taxon index (βNTI) values. The βNTI values were calculated using the null model, where |βNTI| ≥ 2 and |βNTI| < 2 indicated a microbiome assembly predominantly driven by determinism and stochasticity, respectively. The percentages above and below the violin plot represent the proportions of the deterministic processes and stochastic processes, respectively, driving microbiome assembly. **d** Relative contributions of deterministic and stochastic processes to the assembly of phyllosphere epiphytic and endophytic fungal communities in healthy and diseased leaves based on the βNTI values. **e** Relative importance of five ecological processes based on the βNTI and Bray–Curtis-based Raup–Crick index (RCBray) (heterogeneous selection: βNTI < −2; homogeneous selection: βNTI > +2; dispersal limitation: |βNTI| < 2 and RCBray > 0.95; homogenising dispersal: |βNTI| <2 and RCBray < 0.95; and undominated: |βNTI| < 2 and |RCBray| < 0.95).

To investigate the effects of anthracnose on the phyllosphere microbiome of common vetch, we collected leaves from field-grown plants and classified samples as healthy versus anthracnose-symptomatic based on visible symptoms. The results of real-time quantitative polymerase chain reaction (RT-qPCR) showed that the relative abundance of *C. lentis* was significantly higher in leaves with typical lesions compared to healthy leaves (*P* < 0.001), reaching 2.25 × 10^5^ copies/g leaves (Supplementary Fig. 1). Amplicon sequencing was performed to generate microbial community profiles for epiphytic and endophytic bacteria and fungi of healthy and diseased leaves using 16S and ITS metabarcoding data (Supplementary Table 1–4). The microbial communities differed between diseased and healthy leaves. Endophytic bacterial richness was lower in diseased leaves (Wilcoxon; *P* = 0.041), whereas epiphytic bacterial richness showed no significant difference (Wilcoxon; *P* = 0.394). In both niches, diversity (Simpson index) and evenness (Pielou’s evenness) were not significantly affected (Wilcoxon; *P* > 0.05) (Fig. 1a). For fungi, the epiphytic fungal diversity decreased in diseased leaves, while endophytic fungal diversity and evenness increased (Fig. 1b). In contrast to the observed alpha-diversity, principal coordinate analysis (PCoA) confirmed that both epiphytic and endophytic bacterial and fungal communities exhibited distinct beta diversity between health states (PERMANOVA; *P* < 0.01), indicating a pronounced shift in community structure.

Null model analyses revealed that the relative contributions of deterministic and stochastic processes in shaping the assembly of phyllosphere microbial communities were significantly shaped by pathogen infection and compartmental distribution. Disease induced a transition in assembly of the epiphytic bacterial community from predominantly stochastic processes (73%) to predominantly deterministic processes (91%, primarily homogeneous selection) (Fig. 1c, e). By contrast, deterministic processes exerted a stronger influence on endophytic fungal community assembly (87%, primarily homogeneous selection) within diseased leaves (Fig. 1d, e). Overall, disease-mediated deterministic processes exerted the most pronounced effects on leaf epiphytic bacterial communities and endophytic fungal communities. The selective pressures imposed by infection likely promote convergence of epiphytic bacterial communities and deterministic restructuring of endophytic fungal communities within diseased leaves.

### Changes in phyllosphere microbial co-occurrence networks

**Fig. 2:**
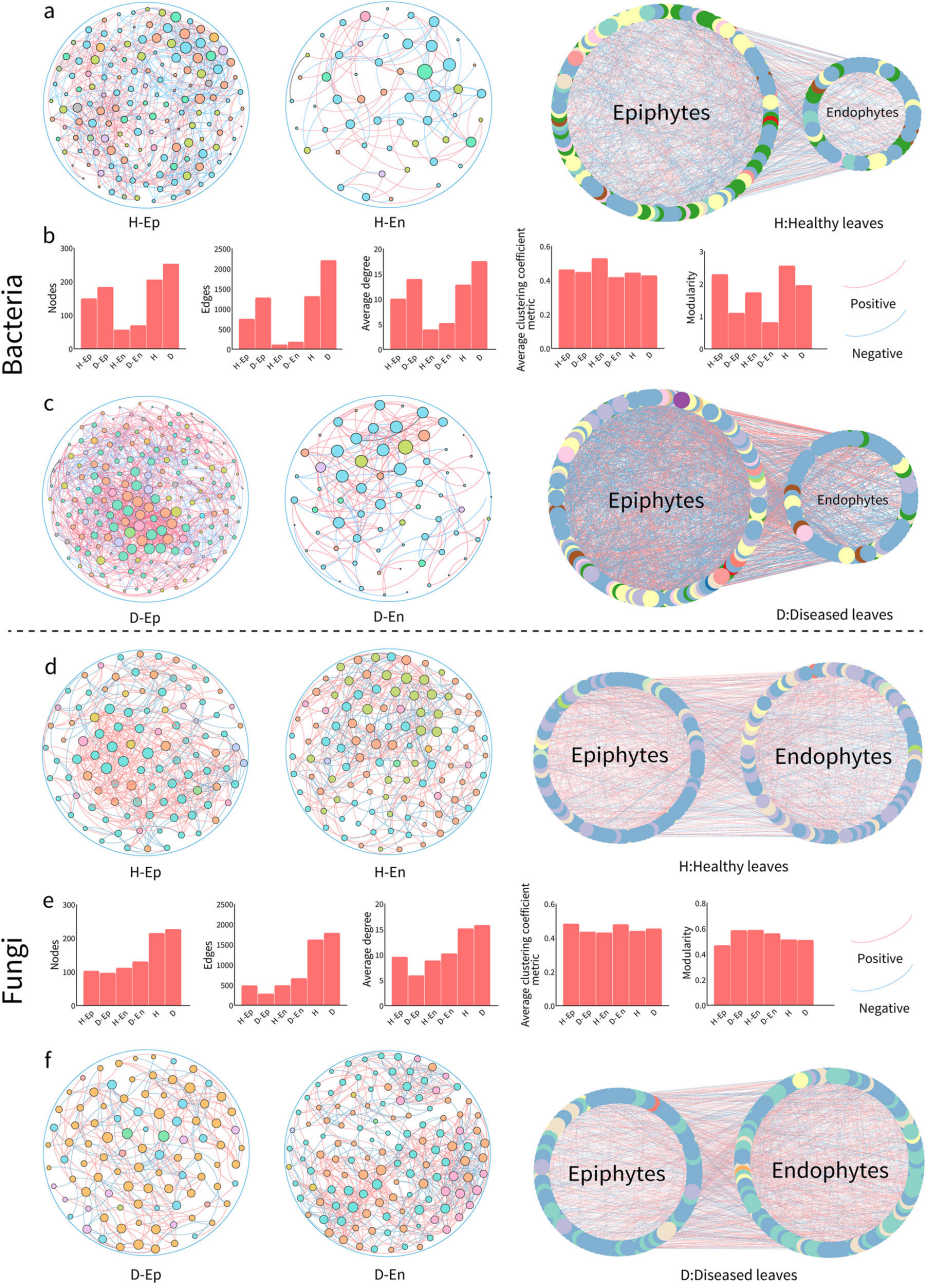
Co-occurrence networks of the phyllosphere microbiomes between healthy and diseased leaves. Network inference was performed based on Spearman correlations at the amplicon sequence variant (ASV) level (abundance > 0.01%, sample prevalence > 1/2) and utilised to compare the bacterial co-occurrence networks of epiphytic and endophytic communities between **a** healthy and **c** diseased leaves. Each node represents a single ASV, where the colour indicates the phylum. Fungal co-occurrence networks of epiphytic and endophytic communities between **d** healthy and **f** diseased leaves (abundance > 0.01%, sample prevalence > 1/2). Network topological properties of **b** bacterial communities and **e** fungal communities. H healthy leaves, D diseased leaves, H-Ep epiphytic microbiome in healthy leaves, H-En endophytic microbiome in healthy leaves, D-Ep epiphytic microbiome in diseased leaves, D-Ep epiphytic microbiome in diseased leaves.

Microbial co-occurrence networks were analysed to compare variations in phyllosphere bacterial and fungal communities and interactions among their members between *C. lentis*-infected leaves and noninfected leaves. For bacteria, network complexity of the epiphytic compartment was greater than that of the endophytic compartment, while network complexity of diseased leaves was greater than that of healthy leaves (Fig. 2a, c). Both epiphytic and endophytic bacterial networks displayed increased complexity following infection. For epiphytes, node counts increased from 149 to 183, the number of edges rose from 748 to 1276, and the average degree was enhanced from 10.04 to 13.945. Endophytes exhibited a similar but weaker trend, with the number of nodes increasing from 56 to 69 and the number of edges from 205 to 252 (Fig. 2b). The increase in network connectivity of bacterial communities was primarily the result of linkages originating from the epiphytic compartment, encompassing interactions such as epiphytic–epiphytic (59.13%) and epiphytic–endophytic (33.15%) connections (Fig. 2a, c). These metric changes confirmed that the pathogen-induced enhancement of network complexity was most pronounced in the epiphytic compartment. By contrast, for fungi, the same network topological properties were not significantly different between healthy and diseased leaves (Fig. 2d–f). Introducing simulated stochastic species loss demonstrated the robustness of the bacterial network. The vulnerability of the bacterial network in the epiphytic compartment and the fungal network in the endophytic compartment increased in diseased leaves compared with healthy ones (Supplementary Fig. 2). In summary, pathogen invasion reshaped the phyllosphere bacterial networks into more complex and interconnected structures, primarily driven by strengthened epiphytic interactions.

### Enrichment of core microorganisms

**Fig. 3:**
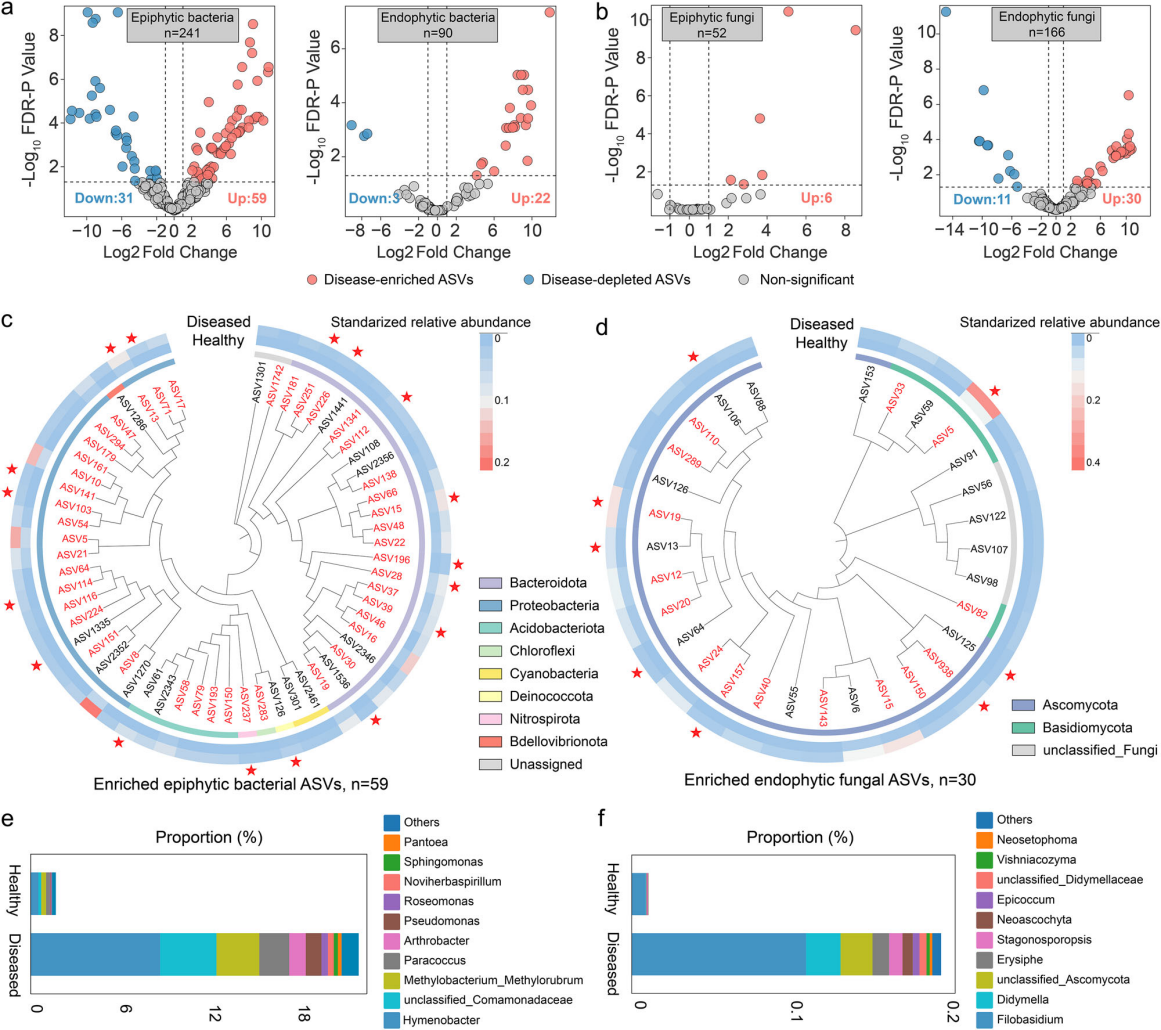
Taxonomic characteristics of differential bacteria and fungi between healthy and diseased phyllosphere microbiotas. Volcano plots depicting amplicon sequence variants (ASVs) enriched or depleted (relative abundance > 0.01%, sample prevalence > 1/2, |log_2_ fold change| ≥ 1, *P* < 0.05) in the **a** epiphytic and endophytic bacteria, as well as the **b** epiphytic fungi and endophytic fungi of diseased samples. Identification of differential epiphytic bacterial ASVs (**c**) and endophytic fungal ASVs (**d**) between healthy and diseased leaves. ASVs highlighted in red display co-occurrence patterns with the pathogen, and red stars denote ASVs that function as keystone hubs within the network. **e** Annotated genera of the enriched epiphytic bacterial ASVs and their proportion. **f** Annotated genera of the enriched endophytic fungal ASVs and their proportion.

Building on the above findings that pathogen infection reshaped phyllosphere microbial community structure and assembly, we further examined the enrichment patterns of core microorganisms across distinct ecological niches to elucidate their roles in disease response. Pathogen infection altered the species composition within the two ecological niches, resulting in many unique ASVs. In the epiphytic compartment, 42.15% of bacterial ASVs and 40.08% of fungal ASVs were detected only in diseased leaves (Supplementary Fig. 3a, b). Similarly, in the endophytic compartment, 51.87% of bacterial ASVs and 41.10% of fungal ASVs were unique to diseased leaves (Supplementary Fig. 3c, d). Differential enrichment analyses showed that bacterial ASVs with relative abundance >0.01% and sample prevalence >1/2 were preferentially enriched in the epiphytic niche (*n* = 59), whereas fungal ASVs were more enriched in the endophytic niche (*n* = 30) (Fig. 3a, b). Within the bacterial endophytic niche, 22 ASVs were significantly enriched, primarily affiliated with the genera *Pantoea* and *Pseudomonas* (Supplementary Fig. 4a, c). Six ASVs with significant differential alterations were detected in the fungal epiphytic niche, among which ASV14 was identified as the *Colletotrichum* pathogen based on sequence alignment (Supplementary Fig. 4b, d).

A total of 59 epiphytic bacterial ASVs were enriched in diseased leaves, which were primarily members of the phyla *Proteobacteria*, *Bacteroidota* and *Actinobacteriota* (Fig. 3a, c). On the genus level, these ASVs were mainly assigned to *Hymenobacter*, *Pantoea*, *Arthrobacter*, *Sphingomonas* and *Pseudomonas* (Fig. 3e and Supplementary Table 5). Among the enriched epiphytic bacterial ASVs, 17 were identified as key nodes within the epiphytic bacterial network under pathogen infection (Fig. 3c and Supplementary Fig. 5), while 45 ASVs exhibited co-occurrence patterns with the pathogenic ASV14 (Fig. 3c and Supplementary Fig. 6a). For endophytic fungi, a total of 30 ASVs were enriched in diseased leaves. These ASVs were mainly assigned to the genera *Filobasidium*, *Erysiphe*, *Neoascochyta*, *Didymella* and *Stagonosporopsis* (Fig. 3d and Supplementary Table 6). Among these enriched endophytic fungal ASVs, eight were identified as key nodes within the endophytic fungal network under pathogen infection (Fig. 3d and Supplementary Fig. 5), while 15 ASVs displayed co-occurrence patterns with the pathogenic ASV45 (Fig. 3d and Supplementary Fig. 6b).

### Alteration of phyllosphere microbial functions in the epiphytic compartment

To complement the amplicon-based analyses, we subsequently applied shotgun metagenomic sequencing to characterise the functional potential of the epiphytic microbiome. Quality filtering and the removal of plant-derived reads yielded 41.6 Gbp of microbial reads. After splicing the sequences, 6,106,386 contigs were produced, with a total length of 420,621 kb for the spliced sequences and an N50 value of 703 bp. After clustering sequences at 95% identity, a total of 5,377,218 nonredundant genes were generated. Further functional annotation of the nonredundant gene set was conducted using general databases such as the Kyoto Encyclopedia of Genes and Genomes (KEGG) and the National Center for Biotechnology Information (NCBI) non-redundant (nr) database, in addition to the specialised Carbohydrate-Active Enzyme (CAZyme) database (Supplementary Table 7). Data on sample species composition and relative abundance were determined based on the species information derived from the sequences aligned to the Nr database. The annotation outcomes revealed that over 80% of the sequences within the epiphytic microbiome in diseased leaves were bacteria, contrasting with only 27.06% in healthy leaves (Supplementary Fig. 7).

**Fig. 4:**
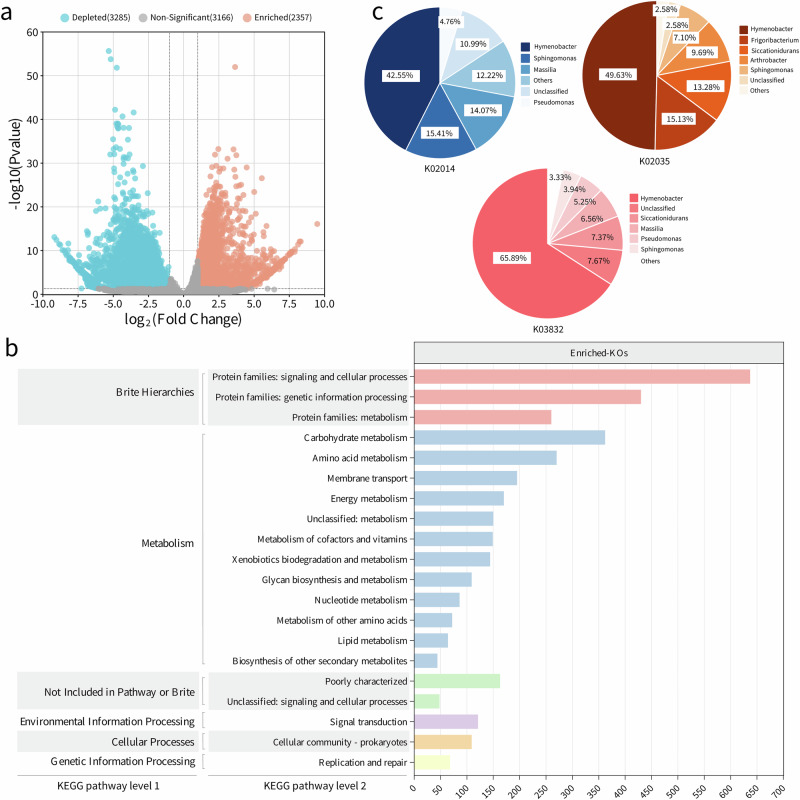
Overview of Kyoto Encyclopedia of Genes and Genomes (KEGG) functional profiling of the bacterial epiphytic microbiome. **a** Enrichment and depletion of KEGG orthologues (KOs), as indicated by fold change >2 or <−2, respectively. **b** Top 20 pathways with enriched KOs in the diseased samples. **c** Taxonomic annotation of KOs from the metagenomic data set.

The KO diversity in leaves infected with *C. lentis* was significantly higher than that in healthy leaves (Kruskal–Wallis test; *P* < 0.01) (Supplementary Fig. 8a). Differential enrichment analyses of the KOs across treatments revealed that 2357 KOs were enriched compared with 3285 KOs that were down-regulated in diseased leaves (Fig. 4a). The KOs enriched in diseased leaves were primarily associated with metabolism-related pathways, comprising carbohydrate metabolism (362 KOs), amino acid metabolism (270 KOs), membrane transport (195 KOs) and energy metabolism (170 KOs) (Fig. 4b). Notably, among the 16 KOs with relative abundance exceeding 0.1%, the ‘iron complex outer membrane receptor protein’ (K02014), was the most differentially enriched KO, with 2.64-fold higher abundance in diseased leaves (Supplementary Table 8). In addition, potential genes associated with bacterial antagonistic disease functions included the ‘basic amino acid/polyamine antiporter, APA family’ (K02035) and the ‘periplasmic protein TonB’ (K03832), with significant enrichment and fold changes of 5.38 and 5.50, respectively. The ‘iron complex outer membrane receptor proteins’ encompassed 6977 contigs from the epiphytic microbiome data set, with 42.55% annotated to *Hymenobacter* spp., 15.41% to *Sphingomonas* spp. and 14.07% to *Pseudomonas* spp. (Fig. 4c). Similarly, ‘periplasmic protein TonB’ consisted of 992 contigs, predominantly annotated as *Hymenobacter* spp. (65.89%), *Sphingomonas* spp. (6.56%) and *Pseudomonas* spp. (5.25%) (Fig. 4c). Further, the ‘basic amino acid/polyamine antiporter, APA family’ consisted of 1087 contigs, with annotations indicating 49.63% as *Hymenobacter* spp., 9.69% as *Sphingomonas* spp. and 7.10% as *Arthrobacter* spp. (Fig. 4c). Other low-abundance taxa were annotated as *Methylobacterium*, *Exiguobacterium*, *Bacillus*, *Pantoea* and *Paracoccus*, among others. Metagenomic sequencing results indicated that the epiphytic microbiome responded to pathogen invasion by competing for iron nutrients.

**Fig. 5:**
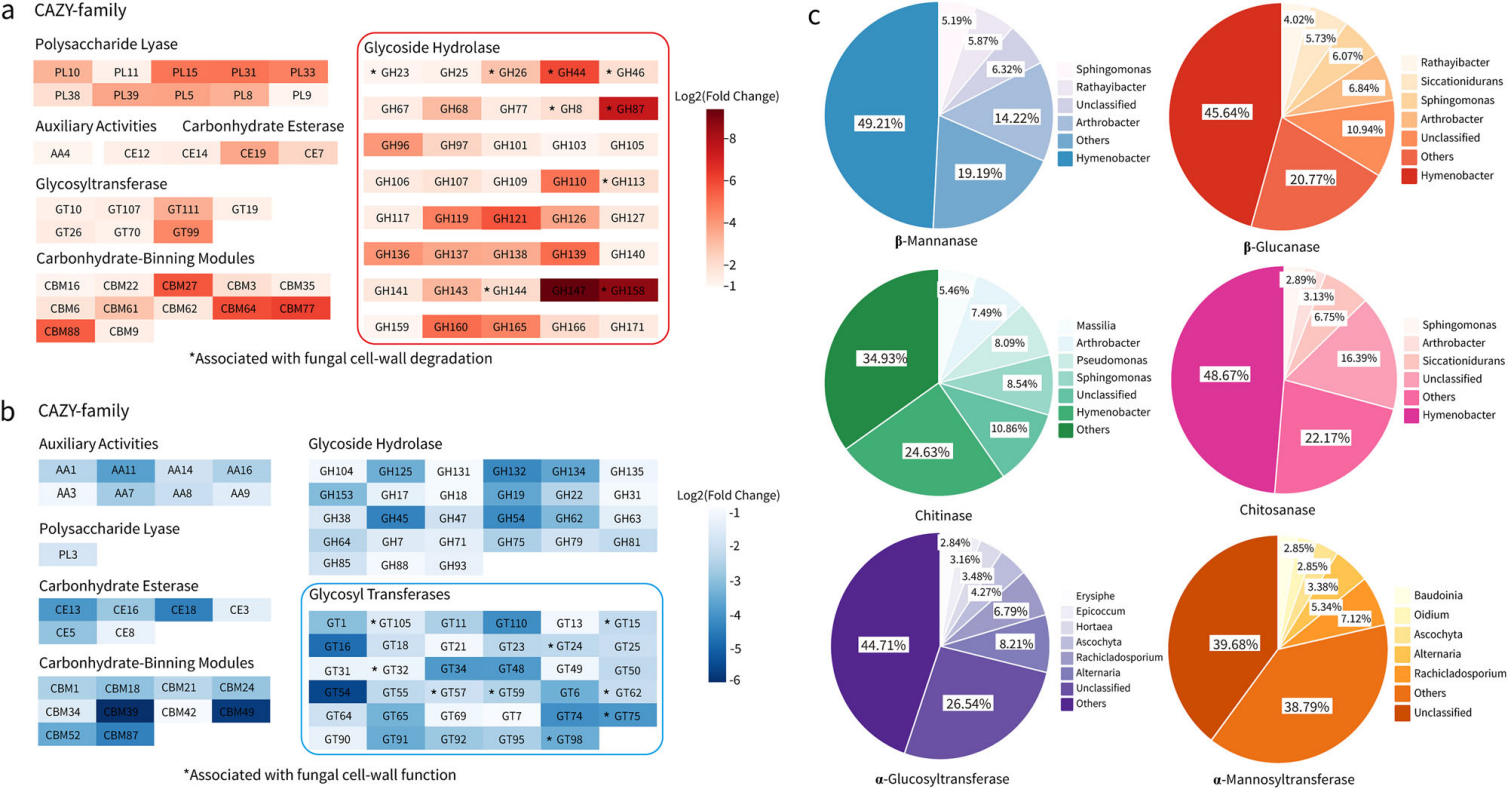
Overview of carbohydrate-active enzymes (CAZymes) functional profiling of the bacterial epiphytic microbiome. **a** Diversity and distribution of enriched CAZymes and **b** depleted CAZymes in the epiphytic microbiome. **c** Taxonomic annotation of CAZyme genes from the metagenomic data set.

Similar to KO diversity, CAZyme diversity was significantly higher in diseased leaves than in healthy leaves (Kruskal–Wallis test; *P* < 0.01) (Supplementary Fig. 8b). In the differential abundance analyses, there were significant variations in 161 CAZyme gene families between healthy and diseased leaves. Compared with healthy leaves 74 CAZyme gene families had higher abundance and 84 had lower abundance in diseased leaves (Fig. 5a). Compared with healthy leaves, the microbiome in diseased leaves had intensified functions of glycoside hydrolases (GHs) (Supplementary Fig. 9), potentially linked to the degradation of fungal cell wall components such as glucans (GH8, GH26, GH44, GH87, GH158 and GH144), mannans (GH26 and GH113), chitosan (GH8 and GH46) and chitinase (GH23). Notably, the GH158 gene family (β-glucanase) was the most enriched, with gene abundance in diseased leaves surpassing that in healthy leaves by a factor of 75.58. The enriched β-glucanase gene family comprised 1187 contig sequences from the epiphytic microbiome data set, predominantly annotated to bacteria, with 45.46% of sequences attributed to *Hymenobacter* spp. and 6.84% to *Arthrobacter* spp. (Fig. 5c). In addition to the high-abundance taxa in the phyllosphere epiphytic community, the low-abundance taxa annotated included *Panobacterium*, *Pantoea*, *Erwinia*, *Pseudomonas* and *Bacillus*.

By contrast, glycosyl transferase (GT) functions declined notably in diseased leaves (Supplementary Fig. 9), potentially linked to the synthesis of fungal cell wall components and functional structures such as α-glucosyltransferases (GT24, GT57, GT59 and GT75) and α- mannosyltransferase transferases (GT15, GT32, GT62 and GT98), among others (Fig. 5b). The α-glucosyltransferase-related gene families encompassed 635 contig sequences, predominantly annotated as fungi, with 8.21% annotated to *Alternaria* spp., 6.79% to *Rachicladosporium* spp. and 0.53% annotated to *Colletotrichum* spp. (Fig. 5c). Notably, the gene family associated with α-mannosyltransferase comprised 1309 contig sequences, with 7.12% annotated to *Rachicladosporium* spp., 5.34% to *Alternaria* spp. and 1.26% annotated to *Colletotrichum* spp. (Fig. 5c). These findings suggest that epiphytic bacteria possess potential antifungal properties, as evidenced by the disease-associated enrichment of genes involved in iron competition (e.g., siderophore transport systems) and fungal cell wall degradation (e.g., glycoside hydrolases) in the epiphytic metagenome.

### Construction of a simplified disease-resistant community

**Fig. 6:**
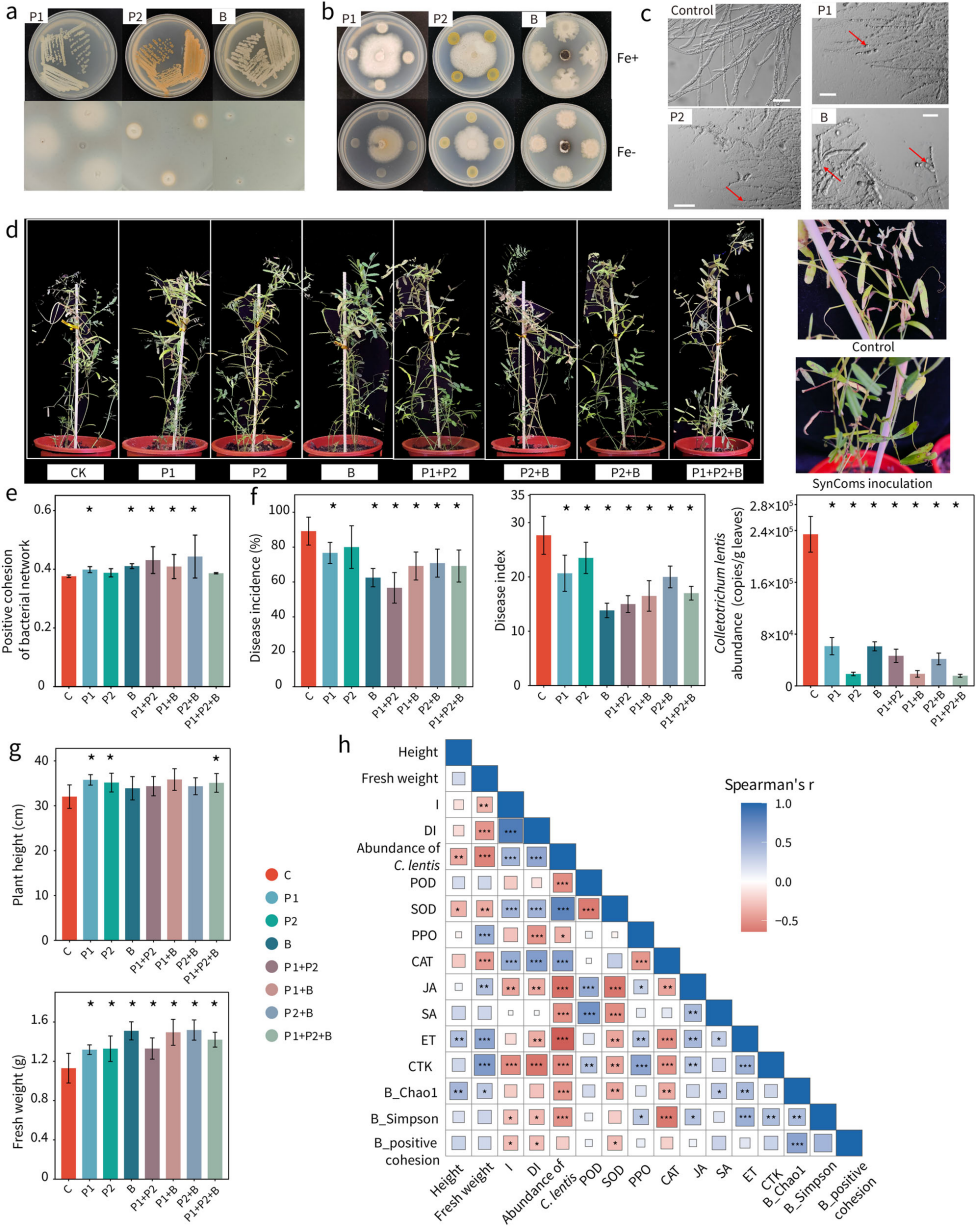
Suppressive activities of selected bacteria that changed in the phyllosphere microbiome against the anthracnose pathogen *Colletotrichum lentis*. **a** Capacity of three bacterial strains to produce siderophores. **b** Inhibitory effects of three bacterial stains on *C. lentis* mycelial growth under iron-rich and iron-limited conditions. **c** Cell wall degradation of crude enzyme extracts from three bacterial strains against pathogens**. d** Plant growth situation and disease suppression of anthracnose treated with different combinations of SynComs in glasshouse experiments. **e** Positive cohesion of the bacterial network following inoculation with different combinations of SynComs. **f** Disease incidence, disease index and the abundance of *C. lentis* of common vetch after inoculation with different combinations of SynComs. **g** Plant height and fresh weight of common vetch post-inoculation with different combinations of SynComs. **h** Spearman correlations between bacterial amplicon sequence variant (ASV) diversity and physiological indicators. Asterisks (*) above bars indicate a significant difference between treatments and the control at *P* < 0.05 based on Student’s *t* test. I disease incidence, DI disease index, SOD superoxide dismutase, CAT catalase, PPO polyphenol oxidase, POD peroxidase, JA jasmonic acid, SA salicylic acid, ET ethylene, CTK cytokinin.

Based on the above identification of core disease-enriched microorganisms and their potential antagonistic functions, we further constructed a simplified synthetic community to evaluate their combined role in conferring resistance against pathogen infection. In a plate-based antagonistic culture assay, 19 bacterial strains isolated from the epiphytic compartment of diseased leaves exhibited varying degrees of antagonistic activity against the pathogen *C. lentis* (Supplementary Fig. 10). Following sequencing data analysis, we selected three isolated epiphytic strains to construct simplified SynComs. Isolated strains were mapped back to ASVs. Sequence alignment of 16S rDNA showed that *Pseudomonas* F12 shared 98.6% sequence similarity with enriched ASV17, while *Pantoea agglomerans* F11 exhibited 98.6% sequence similarity with enriched ASV13. The genome of *Pantoea agglomerans* was reconstructed via metagenomic binning (completeness = 91.67%). This taxon was exclusively detected in the epiphytic compartment of diseased leaves, and its genomic features indicated the capacity for siderophore production and glycoside hydrolase activity (Supplementary Fig. 11 and Supplementary Table 9). Although *Bacillus* B9 did not display significant enrichment in the amplicon sequencing data set, it exhibited the strongest antagonistic activity in vitro, and *Bacillus* was present as a low-abundance but functionally important taxon in the metagenomic data. Evaluation on CAS medium confirmed that all three strains could produce siderophores (Fig. 6a). Notably, under iron-limited conditions, the inhibitory effects of P1 and P2 on *C. lentis* mycelial growth were significantly higher than those under iron-rich conditions (Student’s *t*-test; *P* < 0.05) (Fig. 6b). When *C. lentis* mycelium was treated with crude enzyme extracts, the enzyme extracts from all three epiphytic bacteria degraded fungal cell walls, leading to mycelial rupture (Fig. 6c).

The inhibitory effects of the three bacteria and their synthetic communities on *C. lentis* infection were assessed in live plants (Fig. 6d). The application of various combinations of SynComs altered the community structure of phyllosphere bacteria and fungi (PERMANOVA; *P* = 0.001), increasing the α diversity of phyllosphere bacteria (Supplementary Fig. 12). P1, B and P1 + P2 + B significantly increased the positive cohesion of bacterial networks (*P* < 0.05) (Fig. 6e). Plants sprayed with bacterial suspensions exhibited varying levels of resistance to pathogen infection, as indicated by reductions in the disease index of 12.96% to 44.44% (*P* < 0.05) and in the abundance of *C. lentis* of 73.79% to 93.34% (*P* < 0.05). Nevertheless, disease incidence was not significantly affected in the P2 treatments (*P* > 0.05) (Fig. 6f). In addition, potted plants inoculated with SynComs did not exhibit a significant increase in plant height relative to the control (*P* > 0.05). By contrast, SynCom treatment significantly enhanced plant biomass, with increases ranging from 16.67% to 34.36% compared with the control (*P* < 0.05) (Fig. 6g). SynComs application significantly influenced plant defensive enzyme activity and hormone content. The SynComs significantly reduced SOD and CAT activity (*P* < 0.05), whereas the SynComs P2, P1 + P2, P1 + B, P2 + B and P1 + P2 + B significantly increased leaf peroxidase POD activity compared with that in the control, while SynComs B, P1 + B, P2 + B and P1 + P2 + B significantly increased PPO activity (*P* < 0.05) (Supplementary Fig. 13). The SynComs significantly elevated contents of JA, ET and CTK (*P* < 0.05) (Supplementary Fig. 14). Compared with the control, SynComs P2, P1 + B, P2 + B and P1 + P2 + B significantly increased SA activity (*P* < 0.001) (Supplementary Fig. 14). The Linear mixed model (LLM) analysis revealed that P1 and P2 and their associated SynComs significantly affected the bacterial community postinoculation (*P* < 0.05), whereas B and its associated SynComs significantly influenced plant defensive enzyme activity and hormone levels postinoculation (*P* < 0.05) (Supplementary Table 10). Spearman correlation analysis indicated that PPO activity, JA, ET and CTK contents, the bacterial community Simpson index and positive cohesion were significantly negatively correlated with disease incidence (Fig. 6h). These observations indicate that strains P1 and P2 might mitigate disease by regulating positive interactions within the bacterial community, whereas strain B predominantly suppressed disease by eliciting host defensive responses.

## Discussion

We systematically investigated the differentiation of epiphytic and endophytic microbial niches in common vetch leaves infected by anthracnose and then focused on elucidating the mechanisms by which epiphytic bacteria antagonise the anthracnose pathogen and increase host plant disease resistance. The results provide new insights into the disease-mediated assembly of bacterial epiphytic phyllosphere communities. Notably, the importance of disease-mediated deterministic processes on leaf epiphytic bacterial communities and endophytic fungal communities is a novel observation. This result underscores the heightened susceptibility of open epiphytic ecological niches to bacterial colonisation and interactions following pathogen infection. Furthermore, we found that the disease-induced enrichment of epiphytic bacteria could counteract pathogens through siderophore competition, enzymatic breakdown of fungal cell walls and induction of host immunity. We validated our findings in SynCom inoculation assays and demonstrated the potential of SynComs comprised of beneficial phyllosphere microorganisms to assist plants in combating foliar pathogens.

The responses of phyllosphere microbial communities to pathogen invasion are variable^9,21,22^. In this study, pathogen invasion influenced the assembly of phyllosphere bacterial communities in epiphytic niches and fungal communities in endophytic niches. In addition, we found a shift in community assembly from primarily stochastic processes to primarily deterministic processes, which could be due to niche characteristics, disease-associated environmental filtering, or host effects^23,24^. Bacteria, characterised by rapid reproduction rates and high transmissibility, can swiftly adapt to environmental shifts^25^. Thus, with disease infection, open epiphytic ecological niches were more susceptible to bacterial colonisation and the associated increase in interactions, whereas the endophytic niche predominantly facilitated pathogenic fungal invasions. Moreover, compared with epiphytic fungi, the assembly of phyllosphere endophytic fungi is more host-dependent or host-selective^18,26^, potentially contributing to the shift towards deterministic processes in the assembly of endophytic compartments. Previous observations indicate that internal leaf compartments impose tighter physiological constraints on microbial colonisation^27,28^. Together, these findings highlight fundamental differences in how epiphytic and endophytic microbiomes respond to pathogen invasion.

In contrast to the rhizosphere, the patterns of microbial recruitment and the interactions among phyllosphere microorganisms have yet to be elucidated. We found that the phyllosphere bacteria-to-bacteria microbial network of diseased leaves reflected intense microbial interactions, with the increase in network complexity primarily developing from interactions among epiphytic bacteria. With pathogen invasion, highly interconnected microbiome networks emerge, as observed in diseased leaves, stems and soils^29^. This observation highlights the potential role of mutualistic interactions in increasing community resilience under pathogen stress^30^. Complex microbial network relations have been proposed to enhance plant resistance to pathogen invasion via promoting coordinated interactions, functional complementarity and ecological stability within microbial communities^9,31,32^. Such community-level antagonism represents a characteristic feature of disease-suppressive microbial communities^33^. In contrast to network complexity, robustness decreased and vulnerability increased in networks of epiphytic bacteria and endophytic fungi. Increased network vulnerability suggests reduced redundancy and a greater dependence on key taxa, reflecting a shift toward a specialised but potentially fragile community state^34^. Highly connected microbial networks can be efficient but fragile when dominated by strong interactions and keystone taxa^35^.

Our research shows that disease-induced epiphytic microorganisms can generate siderophores and compete for iron nutrition with a fungal pathogen. Siderophore-producing functional bacteria frequently act as promoters in the rhizosphere and as antagonists against soilborne diseases^36–38^. Iron deficiency significantly increases competitive interactions among strains, with siderophores primarily mediating competition within a colony, whereas competitive production of siderophores notably amplifies the inhibitory effects of *Pseudomonas* sp. on *Ralstonia solanacearum*^39^. In the phyllosphere, iron competition-mediated interactions between bacteria and pathogens are equally effective^40^. We also observed increases in glycoside hydrolases (GHs) in the bacterial microbiome of diseased leaves, which are associated with the breakdown of fungal cell walls. The crude enzyme extract from epiphytic bacteria efficiently degraded the fungal cell walls, rupturing hyphae. This result is supported by research showing that a GH157 β-1,3-glucanase (BsGlc157A) and chitinase inhibit fungal pathogens^41,42^. Moreover, microbial glycoside hydrolases can stimulate plant immunity^43^. With fungal infection of a plant, chitin is hydrolysed, leading to the release of chitin oligosaccharides, which trigger the plant immune response^44^.

The disease-associated enrichment of epiphytic bacteria and their disease-suppressive functions observed in the present research highlight the potential to harness phyllosphere microbiomes for pathogen protection. In the rhizosphere, such disease-induced enrichment of protective microbiota has been well established and can, in some cases, be attributed to a plant-driven ‘cry for help’ response^45,46^. Also for the phyllosphere, Such host-mediated recruitment involving immune signalling^47,48^ and metabolite exudation^49^ has been increasingly proposed. Although the importance of host control remained understudied in the present study, our results raise the question of the extent to which the plant is driving these microbial interactions. Alternative mechanisms may also explain the observed patterns. Pathogen infection can alter phyllosphere conditions, creating niches that favour taxa with traits^50^. Bacteria may respond directly to pathogens or co-disperse as components of a disease-associated microbiome, independent of host influence^51,52^. These processes are not mutually exclusive and reflect broader disease-driven ecological filtering^11,19^.

**Fig. 7:**
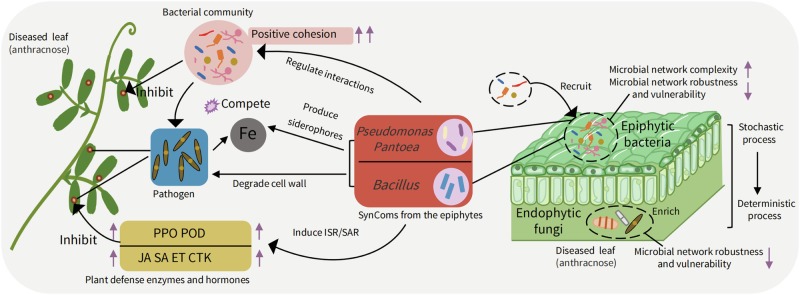
Responses of different ecological niches to pathogen infection and the disease suppression mechanisms of synthetic microbial communities (SynComs). Epiphytic bacteria counteract the pathogen by engaging in siderophore competition and the enzymatic degradation of cell walls. Within SynComs, high-abundance genera in functional clusters, namely *Pseudomonas* and *Pantoea*, regulate bacterial interactions, whereas host immunity responses are induced by low-abundance genera, such as *Bacillus*. PPO polyphenol oxidase, POD peroxidase, JA jasmonic acid, SA salicylic acid, ET ethylene, CTK cytokinin.

SynComs are constructed based on the principles of ecology and synthetic biology and intentionally blend two or more taxonomically identified and functionally distinct microorganisms under precise conditions and defined ratios^53,54^. SynComs provide protection for ungrafted watermelon against *F. oxysporum* via microbial synergistic effects^55^. Additionally, SynComs frequently outperform individual inoculations. A symbiotic community consisting of *S. azotifigens* and *Rhizobium deserti* increases wheat yellow mosaic virus resistance by activating plant hormone-signalling pathways^56^. The validation experiment in this study indicated that the antagonistic capacity of SynComs is a complex synergistic process resulting from competition for iron nutrition between bacteria and pathogens, fungal cell wall degradation, regulation of interactions among members of the phyllosphere bacterial community, and activation of the host immune system (Fig. 7). We observed that high-abundance bacteria inhibited growth of the fungal pathogen while regulating interspecies interactions, whereas low-abundance bacteria activated plant immunity. This finding is consistent with that of previous studies. For example, Li et al.^9^ constructed a simplified SynCom in which the high-abundance bacteria of *Stenotrophomonas* sp., *Rhizobium* sp. and *Ochrobactrum* sp. suppressed pathogen growth, while the low-abundance bacteria of *Advenella* sp. activated plant-induced systemic resistance. These results indicate that SynCom members have distinct functional roles and complementary effects. However, we found that a SynCom with increased complexity is not necessarily the most effective. The number of significant effects suggested that P1 × B was the best SynCom combination, although the combination did not affect plant height and weight. The three-strain combination appeared to be the least effective among the tested treatments. Therefore, a feasible strategy for applications in the phyllosphere is to use a top-down approach that identifies and screens microorganisms with specific functions from a complex natural microbial community to assemble a simplified community with targeted composition and functionality^57,58^.

In conclusion, anthracnose caused a shift in microbiome assembly from stochastic processes to dominance of deterministic processes. The epiphytic domain emerged as the primary site for bacterial enrichment, in contrast to fungal enrichment in the endophytic domain. The disease-triggered surge of epiphytic bacteria might combat pathogens by competing for siderophores, in addition to activating antifungal mechanisms and plant hormone-signalling pathways (Fig. 7). Furthermore, we recommend the use of SynComs in foliar disease management because of the possibility for epiphytic bacteria to confer disease resistance by regulating bacterial interactions and inducing host immunity responses. This study is constrained by its field-based design, which limits causal inference regarding plant–microbe–pathogen interactions. Co-occurrence networks capture statistical associations rather than direct interactions, and the enriched functional potential inferred based on metagenome data reflects the community-level capacity rather than in-situ activity. Moreover, although SynCom isolates were linked to enriched ASVs and functional traits, not all dominant taxa were culturable, and we did not include a cross-kingdom, cross-compartment, or size-matched SynCom composed of non-enriched strains. Future studies integrating metabolomics, host transcriptomics, pathogen-only microcosms and targeted knockout of strain-specific functional genes will be critical to disentangle plant-mediated recruitment from pathogen- or environment-driven selection, thereby enabling a rigorous test of the cry-for-help hypothesis in the phyllosphere.

## Methods

### Sample collection

**Fig. 8:**
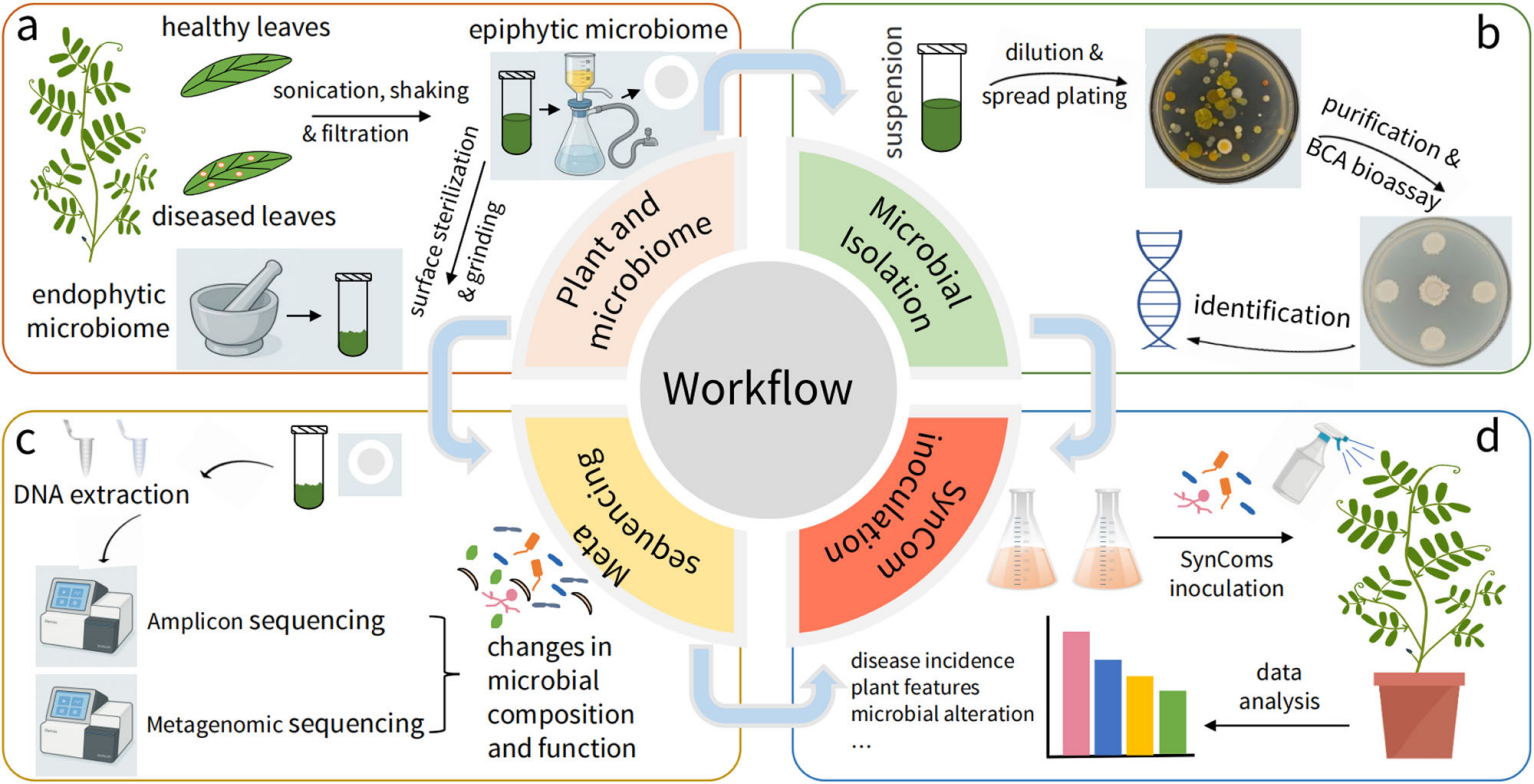
Experimental workflow investigating the impact of a foliar pathogen on phyllosphere microbial communities. **a** Collection of plant leaf samples and separation of epiphytic and endophytic microbiomes. **b** Microbial isolation of epiphytic and endophytic bacteria. **c** Amplicon and metagenomic sequencing of the phyllosphere microbiome. **d** Synthetic microbial community (SynCom) inoculation and evaluation of its antagonistic effect on foliar disease. BCA biological control assay.

In August 2023, two sets of leaf samples were gathered from common vetch fields in Xiahe County, Gansu Province, China (35.22°N, 102.69°E; 2733 m). Initially, visual symptoms were used to categorise the samples as either pathogen-infected (D, diseased leaves) or noninfected (H, healthy leaves). We used a five-point sampling technique and collected samples consisting of 10–15 leaves from each biological replicate, totalling more than 5 g of leaves per sample. Samples were placed in sterile cryopreservation tubes and transported to the laboratory using ice packs, where they were preserved at –80 °C within 24 h for subsequent processing steps. Preparation of epiphytic (Ep) and endophytic (En) samples was based on the methods described by Li et al.^9^ and Yao et al.^18^. To collect the epiphytic (Ep) microbes, leaves from each replicate were initially placed in a 250-mL conical flask containing 200 mL of 0.01 M sterile phosphate-buffered saline (PBS, 4 °C). Subsequently, the leaves underwent a process of sonication (15 min) and shaking (1 h, 200 rpm, 20 °C) to dislodge the epiphytic microbes from the leaf surface into the washing solution. The resultant washing solution was then filtered using a 0.22-μm clean sterile cellulose membrane filter to capture all the microbes present. For the collection of endophytic (En) microbes, the leaf samples, treated as described above, underwent surface sterilisation through sequential immersion for 1 min in 75% ethanol, 3 min in 1% sodium hypochlorite and 30 s in 75% ethanol, followed by three rinses with sterile water. Subsequently, the treated leaves were freeze-dried and homogenised. Four types of samples were prepared, comprising epiphytic and endophytic microbes from healthy (H-Ep and H-En) and diseased leaves (D-Ep and D-En). Six biological replicates were prepared for metabarcoding sequencing and four biological replicates for shotgun metagenomic sequencing for each of the four sample types. The experimental workflow is illustrated in Fig. 8.

### DNA extraction and amplicon sequencing

DNA extraction was performed following the protocol with a Fast DNA SPIN kit (MP Biomedicals, Eschwege, Germany). To amplify the bacterial 16S rRNA gene V3–V4 hypervariable region, primers 338F/806R and 339F/769R were employed, while the fungal ITS1 region was amplified with primers ITS1F/ITS2 (see Supplementary Table 11 for primer details). Subsequent purification of PCR amplicons was conducted and paired-end (250-bp) sequencing was then performed on an Illumina NovaSeq 6000 platform (Illumina, Inc., San Diego, CA, USA). The raw reads underwent quality filtering with Trimmomatic (v0.33)^59^, and primer sequences were trimmed using Cutadapt (v1.9.1)^60^. Denoising was performed to generate ASVs using the Dada2 plugin in QIIME2^61,62^. Finally, the taxonomic classification of ASVs was conducted based on the BLAST alignment of representative sequences against the SILVA (v138) and UNITE (v10.0) databases for bacteria and fungi, respectively^63,64^.

### Metagenomic sequencing

Epiphytic samples were subjected to metagenomic sequencing on an Illumina NovaSeq platform to profile epiphytic microbiome functions. Raw reads were quality-controlled with fastp (v0.22.0)^65^, and host-derived sequences from common vetch were removed by mapping to the reference genome using bowtie2 (v2.1.0)^66^. High-quality reads were assembled using MEGAHIT (v1.2.9)^67^, assemblies were assessed with QUAST (v5.2.0)^68^ and genes were clustered utilising MMseqs2 (v12-113e3)^69^ to generate a nonredundant gene catalogue. Following 95% concordant clustering (≥100 bp), a nonredundant coding gene set was derived from the spliced data set. Clean reads were taxonomically profiled by aligning against the NCBI nr database using Kraken 2^70^. The non-redundant coding gene set underwent comprehensive functional annotation in mainstream databases, including NCBI nr, KEGG and CAZyme.

### **Genome binning**

Assembled metagenomic data were binned with MetaBAT 2, MaxBin 2 and CONCOCT. Refinement and reassembly steps were then performed using the DAS_Tool to combine and improve the results generated by the three binners (completion > 80% and contamination < 10%). The integrated bins were assessed for completeness and contamination using CheckM. High-quality bins were subsequently dereplicated using dRep. We determine the taxonomy of each bin using Taxator-tk against the NCBI nt database. The bins were then quantified by Salmon with default parameters. A phylogenetic tree of bins was constructed using PhyloPhlAn. The genome bins further underwent comprehensive functional annotation in mainstream databases, including NCBI nr, KEGG and CAZyme.

### Bacterial isolation, identification and assessment of antifungal activity

Serial dilutions of suspensions prepared from epiphytic samples were spread on half-strength tryptone soy agar (1/2 TSA) and incubated 30 °C for 48 h. One colony of each morphotype was purified, stored at −80 °C in 50% glycerol and identified using 16S rRNA gene sequences in NCBI-BLAST. The pathogenic fungus causing anthracnose was isolated from diseased leaves and identified morphologically and molecularly as *Colletotrichum lentis* (Supplementary Fig. 15). The in vitro antagonistic activity of phyllosphere bacteria against *C. lentis* was assessed by dual culture assays. A colony of *C. lentis* was positioned at the centre of a potato dextrose agar (PDA) plate. Bacterial suspensions were applied along the plate periphery and then incubated at 28 °C for 10 d. Plates inoculated solely with *C. lentis* were controls. The mycelial growth inhibition rate (R) of *C. lenti* was computed^9^.

To explore the effect of iron nutrition on the activity of the intracellular bacterial antagonist *C. lentis*, in vitro antagonism tests were conducted on the bacteria under both iron-rich and iron-limited conditions^9^. Chrome azurol S (CAS) detection medium was used to evaluate the siderophore production capacity of the phyllosphere bacteria. Furthermore, coculture experiments were performed to evaluate the effect of bacterial metabolites on the growth and development of pathogenic fungal hyphae. The bacterial suspension underwent filtration using a 0.22-μm clean sterile cellulose membrane filter to produce a crude enzyme extract. Subsequently, a sterile cover slip was inserted diagonally into a PDA agar plate, and spinach spores were inoculated at the juncture between the cover slip and the culture medium. The plate was then incubated at 25 °C until the cover slip was completely overlaid with mycelium. Following this, the cover slip was delicately detached and positioned on a microscope slide, with the hyphae-attached side facing upwards. Then, apply 50 μL of the bacterial crude enzyme solution onto the cover slip, transfer the slide into a sterile culture dish, and incubate at 37 °C for 48 h. Analyse the morphological alterations of the hyphae using both a light microscope (Olympus BX-51, Olympus, Tokyo, Japan). Utilise cover slips lacking crude enzyme solution as the control.

### Disease-prevention verification of synthetic microbial communities

A pot experiment was set up to determine whether SynComs have the ability to locally and systemically reduce diseases. The soil mixture used in the experiment was obtained from the field common vetch growth in Xiahe County, Gansu Province, China (35.22°N, 102.69°E, 2,733 m). The mix was composed of 1/3 soil and 2/3 sand. The soil mixture was sterilised in an oven at 169 °C for 24 h. Seeds were surface sterilised by immersion in 1% sodium hypochlorite (NaClO) for 10 min and then rinsed three times with sterile water. The seeds were subsequently immersed in 75% ethanol for 3 min, rinsed three times with sterile water, and then placed on moistened filter paper for germination at 25 °C in the dark. Each pot (12 × 12) contained a total of three common vetch plants. There were six replicates in this experiment, which was conducted in the greenhouse (25 °C and 65% humidity). Daily monitoring and recording of disease symptoms associated with anthracnose were conducted throughout the 7-d pathogen-infection phase, culminating in plant harvest. SynComs were then inoculated three weeks post-planting. Isolated strains were mapped back to ASVs. Among isolated strains, amplicon sequencing data indicated that two strains showed >97% sequence similarity to core-enriched ASVs, including *Pseudomonas* F12 (P1) and *Pantoea agglomerans* F11 (P2). In addition, *Bacillus* F9 (B), which exhibited the strongest inhibitory activity in the in-vitro antagonistic assays, was also included in the SynCom. According to the metagenomic data, *Bacillus* was present as a low-abundance but functionally important taxon. We conducted a pot experiment with the following treatment groups: (i) control, inoculated with 1 mL of sterile water; (ii) P1, P2 and B, with each individually inoculated with 1 mL of suspension; (iii) P1 + P2, P1 + B and P2 + B, with pairwise inoculation with 1 mL of suspension; and (iv) P1 + P2 + B, with co-inoculation with 1 mL of suspension. In the SynCom treatments, equal volumes of P1, P2 and B suspensions were combined in various combinations, with a total of 1 mL suspension. The OD_600_ was adjusted to 0.6^71^. Each individual treatment contained six pots, totalling 48 pots. Following a 2-d interval, *C. lentis* spore suspensions were applied to the leaves of common vetch. The pathogen was cultured on PDA plates, and the conidia were collected to prepare the pathogen inoculum. A suspension containing 1 × 10^6^ conidia/mL was sprayed on common vetch. Following pathogen application, the plants were covered with a black plastic enclosure for 72 h to maintain optimal humidity levels. Daily monitoring and recording of disease symptoms associated with anthracnose were conducted throughout the 7-d pathogen-infection phase, culminating in plant harvest. The incidence rate (I) and disease index (DI) were computed^21^:

$$I( \% )=\frac{{\rm{number}}\,{\rm{of}}\,{\rm{diseased}}\,{\rm{leaves}}}{{\rm{total}}\,{\rm{number}}\,{\rm{of}}\,{\rm{leaves}}\,{\rm{investigated}}}\times 100$$

$${\rm{DI}}=\frac{\sum ({\rm{class}}\,{\rm{frequency}}\times {\rm{disease}}\,{\rm{severity}}\,{\rm{scale}})}{({\rm{total}}\,{\rm{number}}\,{\rm{of}}\,{\rm{leaves}}\,{\rm{investigated}})\times 5}\,\times 100$$

The following disease severity scale of 0 to 5 was used: 0 = healthy, 1 = 1% to 10% of branch area affected, 2 = 11% to 25% of branch area affected, 3 = 26% to 50% of branch area affected, 4 = 51% to 75% of branch area affected and 5 = 76% to 100% of branch area affected.

Plant height and fresh weight were then determined. Fresh leaves from each common vetch plant in the pots were harvested to measure peroxidase (POD), superoxide dismutase (SOD), catalase (CAT) and polyphenol oxidase (PPO) levels through colourimetric assay kits (Solarbio Technology Co., Ltd, Beijing, China). Plant phytohormone levels, including ethylene (ET), jasmonic acid (JA), salicylic acid (SA) and cytokinin (CTK), were determined using enzyme-linked immunosorbent assay (ELISA) kits (Yuanjie Biotechnology Centre, Shanghai, China) based on the double-antibody sandwich method. In addition, 5–10 randomly selected leaves per pot were utilised for amplicon sequencing.

### RT-qPCR amplifications

The abundance of *C. lentis* in common vetch leaves was determined via RT-qPCR. The total DNA was extracted from leaf samples weighing 0.2 g using a Fast DNA SPIN Kit (Omega Bio-Tek Inc., Norcross, GA, USA) according to the manufacturer’s instructions. We designed the primer pair CL1F/CL1R based on the genomic DNA sequence NWBT00000000 on the NCBI for *C. lentis* to amplify its internal transcribed spacer (ITS) region^72^. The primer sequence is shown in Supplementary Table 11. RT-qPCR was performed in a 20-μL reaction mixture composed of 10 μL TB Green Premix Ex Taq II (2×) (TaKaRa Biomedical Technology Co. Ltd., Dalian, China), 0.8 μL of each forward and reverse primer, 2 μL of DNA and 6.4 μL of ddH_2_O. The thermal conditions for the PCR reaction were as follows: 95 °C for 3 min, 95 °C for 30 s, 55 °C for 30 s, 72 °C for 30 s and 40 cycles. To confirm the specificity of amplification, melting curve analysis and gel electrophoresis were conducted. A standard curve was generated using plasmid DNA, and the abundance of *C. lentis* was calculated based on this standard curve. The results were expressed as the log_10_ values of the number of gene copies per gram of leaf sample.

### Statistical analyses

All analyses were conducted using R software (v4.3.2) unless stated otherwise. The ‘vegan’ package was used to calculate alpha diversity indices and beta diversity according to principal component analysis (PCA) based on Bray–Curtis distance. Alpha diversity of the phyllosphere microbiotas was assessed through calculations of richness (Chao1 index), diversity (Simpson index) and evenness (Pielou’s evenness). PERMANOVA was used to test differences in epiphytic and endophytic microbial communities indicated by PCA. The shared ASVs were analysed with Venn diagrams using the ‘VennDiagram’ package. A prevalence-based filtering strategy was applied to remove low-prevalence ASVs detected in only a small number of samples, thereby reducing data sparsity and minimising the influence of spurious sequences^73,74^. For each group of samples, ASVs present in more than 1/2 of the samples that displayed a relative abundance ≥ 0.01% were selected for analysis. Differential abundance analysis of ASVs was performed in edgeR, with differences considered significant at a *P* < 0.05 and |log_2_ fold-change| ≥ 1. Co-occurrence networks were inferred using Spearman correlations at the ASV and genus levels (abundance > 0.01%, sample occupancy rate > 1/2). Edges were established for |*r*| ≥ 0.8 with false discovery rate (FDR)-adjusted *P* < 0.05. Network topology was quantified in R using igraph (v1.2.6). Figures of networks were prepared in Gephi (v9.0) and Cytoscape (v3.10.3). To identify potential core species in the network, we calculated the within-module connectivity (*Zi*) and among-module connectivity (*Pi*) of each node. We then identified module hubs (Zi ≥ 2.5, Pi < 0.62), connectors (Zi < 2.5, Pi ≥ 0.62) and network hubs (Zi ≥ 2.5, Pi ≥ 0.62), which were referred to as keystone nodes^75^. Moreover, the stability of the microbial co-occurrence networks was evaluated using multiple indices. Robustness was simulated by randomly removing a progressively increasing proportion of nodes^76^. Vulnerability was determined as the maximum reduction in network efficiency following the deletion of any single node^76^. The community connectivity was quantified as cohesion, according to the method of Herren and McMahon^77^. We computed the beta nearest taxon index (βNTI) based on a null model (999 randomisations) to evaluate the contributions of deterministic (|βNTI| ≥ 2) versus stochastic processes (|βNTI| < 2) to microbiome assembly^78^.

Statistical analyses included nonparametric Kruskal–Wallis tests used to compare healthy and diseased groups and Student’s *t* tests to evaluate differences in plant growth, physiology and phyllosphere microbiota between the control and various SynCom inoculation groups. The *P*-values were adjusted for multiple comparisons using the Benjamini–Hochberg FDR method. Associations between microbial communities and plant properties were inferred by using Spearman correlations in the ‘corrplot’ package. LLMs were used to examine the effects of different SynCom combinations on plant growth indicators, defensive enzyme activity and hormones. The three bacteria introduced for inoculation were considered as fixed effects, while replicates (pots) were regarded as random effects. The model was fit using the lmer function from the lme4 package.

## Supplementary information

Supplementary Information.

## Data Availability

The raw sequencing data generated in this study have been deposited in the NCBI BioProject database under accession numbers PRJNA1303966 and PRJNA1303968 (amplicon sequencing) and PRJNA1303619 (metagenome sequencing). All source data files are available from the corresponding author upon reasonable request.
