# Supplementary material for: Anthracnose drives assembly of phyllosphere epiphytic bacterial communities to increase disease resistance
Source: NPJ Biofilms Microbiomes. 2026 May 15;12:145. doi: 10.1038/s41522-026-00991-z (PMC13429687; doi:10.1038/s41522-026-00991-z)
Supplement: Supplementary file 1 — Supplementary Information. [file 41522_2026_991_MOESM1_ESM.pdf]

Supplementary Information for

Anthracnose drives assembly of phyllosphere epiphytic bacterial communities to increase disease resistance

Rongchun Zheng[1,2,3,4], Yingde Li[1,2,3,4], Panpan Shang[1,2,3,4], Youlei Shen[1,2,3,4], Zhibiao Nan[1,2,3,4]*, Tingyu Duan[1,2,3,4]*

[1]State Key Laboratory of Herbage Improvement and Grassland Agro-ecosystems, Lanzhou University, Lanzhou 730000, China; Key Laboratory of Grassland Livestock Industry Innovation, Ministry of Agriculture and Rural Affairs, Lanzhou 730000, China; Engineering Research Center of Grassland Industry, Ministry of Education, Gansu Tech Innovation Centre of Western China Grassland Industry, Lanzhou 730000, China; College of Pastoral Agriculture Science and Technology, Lanzhou University, Lanzhou 730000, China.

*Corresponding author:

T.Y. Duan and

Z.B. Nan

Supplementary Tables

Supplementary Table 1 Overview of sequencing data for phyllosphere epiphytic bacteria

Sample ID	Raw reads	Clean reads	Denoised reads	Merged reads	Non-chimeric reads	ASV Numbers
D-Ep1	80189	76062	75810	72911	58900	822
D-Ep 2	79823	75816	75481	72833	63190	917
D-Ep 3	79908	75842	75558	72755	62280	1021
D-Ep 4	80009	76087	75809	73103	62219	920
D-Ep 5	79989	75665	75417	74057	67669	921
D-Ep 6	80177	75377	75085	71963	58380	731
H-Ep 1	80146	76251	75925	74214	69376	861
H-Ep 2	79934	76072	75874	74544	69633	691
H-Ep 3	79968	75737	75557	74062	69221	967
H-Ep 4	79924	76261	76107	74473	70891	1020
H-Ep 5	79972	75712	75462	73769	69954	942
H-Ep 6	79715	75790	75577	73824	69628	1136

Supplementary Table 2 Overview of sequencing data for phyllosphere endophytic bacteria

Sample ID	Raw reads	Clean reads	Denoised reads	Merged reads	Non-chimeric reads	ASV Numbers
D-En1	79968	74799	74232	65702	46516	854
D-En 2	79931	75281	74649	66819	50315	832
D-En 3	79949	75148	74515	63580	47809	883
D-En 4	79976	72984	71995	66073	54737	768
D-En 5	79871	72582	72319	68976	48035	717
D-En 6	79750	74549	74058	65892	47456	757
H-En 1	80075	76205	75801	69466	56313	909
H-En 2	79799	75742	75305	67760	55910	991
H-En 3	79789	75470	74885	65294	52365	895
H-En 4	80139	75996	75501	66580	55080	901
H-En 5	79620	75596	75060	68283	60649	955
H-En 6	79840	76429	75934	67752	56116	728

Supplementary Table 3 Overview of sequencing data for phyllosphere epiphytic fungi

Sample ID	Raw reads	Clean reads	Denoised reads	Merged reads	Non-chimeric reads	ASV Numbers
D-Ep1	80016	75141	75010	73436	72318	651
D-Ep 2	79754	75039	74942	73365	72363	647
D-Ep 3	80171	75050	74948	73569	72183	753
D-Ep 4	79991	76009	75894	74216	73329	709
D-Ep 5	79883	75709	75585	74009	72981	652
D-Ep 6	79712	75071	74971	73891	72831	581
H-Ep 1	79887	75555	75413	73939	73230	633
H-Ep 2	80260	75086	74925	73519	72782	644
H-Ep 3	80246	75299	75200	73414	72628	830
H-Ep 4	79976	75015	74934	73325	72227	745
H-Ep 5	80123	75848	75721	74384	73392	629
H-Ep 6	79957	74595	74494	73257	72482	622

Supplementary Table 4 Overview of sequencing data for phyllosphere endophytic fungi

Sample ID	Raw reads	Clean reads	Denoised reads	Merged reads	Non-chimeric reads	ASV Numbers
D-En1	480736	418050	417430	416254	375532	492
D-En 2	469665	411171	410028	408283	398529	429
D-En 3	479721	409729	409391	408657	371002	610
D-En 4	479950	409347	408766	407594	378500	524
D-En 5	479610	415108	414809	414063	386091	647
D-En 6	479892	412215	411599	410988	396762	392
H-En 1	479858	415171	414338	412771	400988	574
H-En 2	480881	420424	420241	419661	404274	319
H-En 3	479383	415485	414735	413875	391061	681
H-En 4	480659	416457	415780	414302	396635	560
H-En 5	480039	412614	412068	410790	397435	407
H-En 6	480061	418169	417644	416750	394331	330

Supplementary Table 5 Enriched ASVs of epiphytic bacteria and their taxonomic annotations

	logFC	FDR	PValue	Phylum	Class	Order	Family	Genus
ASV79	9.126327	6.64E-05	8.26E-06	Actinobacteriota	Actinobacteria	Micrococcales	Microbacteriaceae	Rathayibacter
ASV58	3.735364	0.037196	0.013582	Actinobacteriota	Actinobacteria	Micrococcales	Micrococcaceae	Arthrobacter
ASV61	10.75463	4.71E-07	1.96E-08	Actinobacteriota	Actinobacteria	Micrococcales	Micrococcaceae	Arthrobacter
ASV2343	9.730674	5.23E-05	5.78E-06	Actinobacteriota	Actinobacteria	Micrococcales	Micrococcaceae	Arthrobacter
ASV193	8.931251	6.32E-08	1.84E-09	Actinobacteriota	Actinobacteria	Frankiales	Nakamurellaceae	Nakamurella
ASV150	8.679018	2.06E-08	5.14E-10	Actinobacteriota	Actinobacteria	Propionibacteriales	Nocardioidaceae	Marmoricola
ASV179	4.433488	0.001598	0.000378	Proteobacteria	Alphaproteobacteria	Acetobacterales	Acetobacteraceae	Roseomonas
ASV47	2.939152	0.023389	0.007764	Proteobacteria	Alphaproteobacteria	Acetobacterales	Acetobacteraceae	Roseomonas
ASV294	2.964636	0.023576	0.007924	Proteobacteria	Alphaproteobacteria	Acetobacterales	Acetobacteraceae	Rubritepida
ASV5	2.982295	0.000275	4.56E-05	Proteobacteria	Alphaproteobacteria	Rhizobiales	Beijerinckiaceae	Methylobacterium_Methylorubrum
ASV21	2.306612	0.009793	0.002704	Proteobacteria	Alphaproteobacteria	Rhizobiales	Beijerinckiaceae	Methylobacterium_Methylorubrum
ASV141	7.276401	1.76E-06	9.49E-08	Proteobacteria	Alphaproteobacteria	Caulobacterales	Caulobacteraceae	Brevundimonas
ASV161	7.432907	2.52E-05	1.98E-06	Proteobacteria	Alphaproteobacteria	Rhizobiales	Rhizobiaceae	Aureimonas
ASV10	2.5318	0.00136	0.000305	Proteobacteria	Alphaproteobacteria	Rhodobacterales	Rhodobacteraceae	Paracoccus
ASV103	7.808976	0.000162	2.42E-05	Proteobacteria	Alphaproteobacteria	Sphingomonadales	Sphingomonadaceae	unclassified_Sphingomonadaceae
ASV54	3.490885	0.03868	0.014284	Proteobacteria	Alphaproteobacteria	Sphingomonadales	Sphingomonadaceae	Sphingomonas
ASV181	7.787615	2.77E-07	1.04E-08	Bacteroidota	Bacteroidia	Chitinophagales	Chitinophagaceae	Flavisolibacter
ASV226	6.65029	7.78E-05	1.03E-05	Bacteroidota	Bacteroidia	Chitinophagales	Chitinophagaceae	Segetibacter
ASV251	5.91811	0.000293	5.34E-05	Bacteroidota	Bacteroidia	Chitinophagales	Chitinophagaceae	Flavisolibacter
ASV138	2.46345	0.036378	0.012981	Bacteroidota	Bacteroidia	Cytophagales	Hymenobacteraceae	Hymenobacter
ASV19	2.680493	0.017184	0.005633	Bacteroidota	Bacteroidia	Cytophagales	Hymenobacteraceae	Hymenobacter
ASV196	3.149812	0.035038	0.012358	Bacteroidota	Bacteroidia	Cytophagales	Hymenobacteraceae	Hymenobacter
ASV28	2.07774	0.0149	0.004575	Bacteroidota	Bacteroidia	Cytophagales	Hymenobacteraceae	Hymenobacter
ASV16	4.978396	0.002393	0.000606	Bacteroidota	Bacteroidia	Cytophagales	Hymenobacteraceae	Hymenobacter
ASV2356	7.53759	0.000229	3.61E-05	Bacteroidota	Bacteroidia	Cytophagales	Hymenobacteraceae	Hymenobacter
ASV22	4.195132	0.00136	0.000316	Bacteroidota	Bacteroidia	Cytophagales	Hymenobacteraceae	Hymenobacter
ASV48	4.231337	0.012995	0.003882	Bacteroidota	Bacteroidia	Cytophagales	Hymenobacteraceae	Hymenobacter
ASV112	4.954521	0.000457	8.73E-05	Bacteroidota	Bacteroidia	Cytophagales	Hymenobacteraceae	Adhaeribacter
ASV1341	6.379433	0.000162	2.29E-05	Bacteroidota	Bacteroidia	Cytophagales	Hymenobacteraceae	Adhaeribacter
ASV15	4.330454	0.00136	0.000313	Bacteroidota	Bacteroidia	Cytophagales	Hymenobacteraceae	Hymenobacter
ASV39	2.683999	0.001786	0.00043	Bacteroidota	Bacteroidia	Cytophagales	Hymenobacteraceae	Hymenobacter
ASV30	3.935836	0.012204	0.003586	Bacteroidota	Bacteroidia	Cytophagales	Hymenobacteraceae	Hymenobacter

ASV37	4.364675	0.034295	0.011953	Bacteroidota	Bacteroidia	Cytophagales	Hymenobacteraceae	Hymenobacter
ASV46	10.80865	2.77E-07	9.42E-09	Bacteroidota	Bacteroidia	Cytophagales	Hymenobacteraceae	Hymenobacter
ASV66	6.492532	0.002622	0.000685	Bacteroidota	Bacteroidia	Cytophagales	Hymenobacteraceae	Hymenobacter
ASV1536	5.510585	0.001828	0.000448	Bacteroidota	Bacteroidia	Cytophagales	Hymenobacteraceae	Hymenobacter
ASV2346	9.454462	5.23E-05	5.56E-06	Bacteroidota	Bacteroidia	Cytophagales	Hymenobacteraceae	Hymenobacter
ASV1441	5.324875	0.002332	0.000581	Bacteroidota	Bacteroidia	Cytophagales	Hymenobacteraceae	Pontibacter
ASV108	4.256343	0.009793	0.002732	Bacteroidota	Bacteroidia	Cytophagales	Hymenobacteraceae	Hymenobacter
ASV283	5.361462	0.00262	0.000674	Chloroflexi	Chloroflexia	Thermomicrobiales	AKYG1722	uncultured_Alphaproteobacteria_bac
ASV301	6.289496	0.00085	0.000176	Cyanobacteria	Cyanobacteriia	Phormidesmiales	Nodosilineaceae	Nodosilinea_PCC_7104
ASV2461	5.78983	0.00136	0.000302	Cyanobacteria	Cyanobacteriia	Oxyphotobacteria_Incertae_Sedis	Unknown_Family	Leptolyngbya_EcFYyyy_00
ASV126	3.751731	0.014727	0.004461	Deinococcota	Deinococci	Deinococcales	Deinococcaceae	Deinococcus
ASV8	3.964467	1.10E-05	7.30E-07	Proteobacteria	Gammaproteobacteria	Burkholderiales	Comamonadaceae	unclassified_Comamonadaceae
ASV1270	7.372254	0.000281	4.78E-05	Proteobacteria	Gammaproteobacteria	Burkholderiales	Comamonadaceae	unclassified_Comamonadaceae
ASV17	10.20795	7.75E-05	9.97E-06	Proteobacteria	Gammaproteobacteria	Enterobacterales	Erwiniaceae	Pantoea
ASV151	7.456935	0.000256	4.14E-05	Proteobacteria	Gammaproteobacteria	Burkholderiales	Oxalobacteraceae	Massilia
ASV2352	8.556031	7.83E-05	1.07E-05	Proteobacteria	Gammaproteobacteria	Burkholderiales	Oxalobacteraceae	Massilia
ASV64	9.533778	1.18E-06	5.41E-08	Proteobacteria	Gammaproteobacteria	Burkholderiales	Oxalobacteraceae	Noviherbaspirillum
ASV114	9.027829	3.00E-09	6.22E-11	Proteobacteria	Gammaproteobacteria	Burkholderiales	Oxalobacteraceae	Noviherbaspirillum
ASV116	4.546804	0.010312	0.002952	Proteobacteria	Gammaproteobacteria	Burkholderiales	Oxalobacteraceae	Noviherbaspirillum
ASV1335	7.10176	0.000293	5.36E-05	Proteobacteria	Gammaproteobacteria	Burkholderiales	Oxalobacteraceae	Noviherbaspirillum
ASV224	7.177833	5.07E-05	4.92E-06	Proteobacteria	Gammaproteobacteria	Burkholderiales	Oxalobacteraceae	unclassified_Oxalobacteraceae
ASV13	4.076145	0.016991	0.005464	Proteobacteria	Gammaproteobacteria	Pseudomonadales	Pseudomonadaceae	Pseudomonas
ASV71	8.533677	0.000162	2.36E-05	Proteobacteria	Gammaproteobacteria	Enterobacterales	unclassified_Enterobacterales	unclassified_Enterobacterales
ASV237	5.926576	0.00114	0.000241	Nitrospirota	Nitrospiria	Nitrospirales	Nitrospiraceae	Nitrospira
ASV1286	7.742732	2.52E-05	1.81E-06	Bdellovibrionota	Oligoflexia	Oligoflexales	Oligoflexaceae	Oligoflexus
ASV1301	6.615506	0.000473	9.41E-05	Unassigned	Unassigned	Unassigned	Unassigned	Unassigned
ASV1742	3.843482	0.0236	0.00803	Unassigned	Unassigned	Unassigned	Unassigned	Unassigned

Supplementary Table 6 Enriched ASVs of endophytic fungi and their taxonomic annotations

	logFC	FDR	PValue	Phylum	Class	Order	Family	Genus
ASV143	4.361396	0.022006	0.004364	Ascomycota	Eurotiomycetes	Chaetothyriales	Trichomeriaceae	Knufia
ASV6	4.427164	0.045506	0.0109	Ascomycota	Leotiomycetes	Erysiphales	Erysiphaceae	Erysiphe
ASV125	7.565514	0.000411	3.44E-05	Ascomycota	Sordariomycetes	Glomerellales	Plectosphaerellaceae	Lectera
ASV150	9.511744	0.000619	7.04E-05	Ascomycota	Dothideomycetes	Mycosphaerellales	Mycosphaerellaceae	unclassified_Mycosphaerellaceae
ASV938	10.71739	0.000353	2.75E-05	Ascomycota	Dothideomycetes	Mycosphaerellales	Mycosphaerellaceae	unclassified_Mycosphaerellaceae
ASV126	4.999554	0.031682	0.007117	Ascomycota	Dothideomycetes	Pleosporales	Pleosporaceae	Alternaria
ASV88	9.600978	9.44E-05	2.83E-06	Ascomycota	Dothideomycetes	Pleosporales	Pleosporaceae	Comoclathris
ASV19	6.029546	0.003382	0.000527	Ascomycota	Dothideomycetes	Pleosporales	Didymellaceae	Didymella
ASV13	5.419247	0.032119	0.007501	Ascomycota	Dothideomycetes	Pleosporales	Didymellaceae	Epicoccum
ASV24	4.50494	0.007707	0.001338	Ascomycota	Dothideomycetes	Pleosporales	Didymellaceae	Neoascochyta
ASV157	8.826646	0.000583	6.28E-05	Ascomycota	Dothideomycetes	Pleosporales	Didymellaceae	Neoascochyta
ASV106	8.284028	0.000627	7.51E-05	Ascomycota	Dothideomycetes	Pleosporales	Pleosporaceae	Neocamarosporium
ASV64	8.632668	0.000732	9.20E-05	Ascomycota	Dothideomycetes	Pleosporales	Didymellaceae	Neodidymelliopsis
ASV40	11.93444	3.03E-07	5.44E-09	Ascomycota	Dothideomycetes	Pleosporales	Phaeosphaeriaceae	Neosetophoma
ASV55	4.140442	0.031682	0.007057	Ascomycota	Dothideomycetes	Pleosporales	Phaeosphaeriaceae	Paraphoma
ASV12	2.911339	0.031682	0.006802	Ascomycota	Dothideomycetes	Pleosporales	Didymellaceae	Stagonosporopsis
ASV20	4.179515	0.022006	0.00448	Ascomycota	Dothideomycetes	Pleosporales	Didymellaceae	unclassified_Didymellaceae
ASV110	10.45232	0.000419	3.76E-05	Ascomycota	Dothideomycetes	Pleosporales	Leptosphaeriaceae	unclassified_Leptosphaeriaceae
ASV289	6.302489	0.006155	0.001023	Ascomycota	Dothideomycetes	Pleosporales	unclassified_Pleosporales	unclassified_Pleosporales
ASV153	8.169929	0.000242	1.45E-05	Ascomycota	Taphrinomycetes	Taphrinales	Protomycetaceae	Protomyces
ASV15	7.782753	0.001109	0.000166	Ascomycota	unclassified_Ascomycota	unclassified_Ascomycota	unclassified_Ascomycota	unclassified_Ascomycota
ASV82	9.856941	4.69E-05	1.12E-06	Basidiomycota	Tremellomycetes	Cystofilobasidiales	Cystofilobasidiaceae	Cystofilobasidium
ASV5	2.860534	0.022006	0.004302	Basidiomycota	Tremellomycetes	Filobasidiales	Filobasidiaceae	Filobasidium
ASV59	10.11246	0.000321	2.31E-05	Basidiomycota	Tremellomycetes	Filobasidiales	Filobasidiaceae	Filobasidium
ASV33	4.254591	0.031682	0.007209	Basidiomycota	Tremellomycetes	Tremellales	Bulleribasidiaceae	Vishniacozyma
ASV91	9.149833	0.000488	4.92E-05	Basidiomycota	unclassified_Basidiomycota	unclassified_Basidiomycota	unclassified_Basidiomycota	unclassified_Basidiomycota
ASV56	10.5098	0.000243	1.60E-05	unclassified_Fungi	unclassified_Fungi	unclassified_Fungi	unclassified_Fungi	unclassified_Fungi
ASV98	8.847003	0.000488	4.97E-05	unclassified_Fungi	unclassified_Fungi	unclassified_Fungi	unclassified_Fungi	unclassified_Fungi
ASV107	8.082262	0.000826	0.000114	unclassified_Fungi	unclassified_Fungi	unclassified_Fungi	unclassified_Fungi	unclassified_Fungi
ASV122	8.127742	0.000852	0.000122	unclassified_Fungi	unclassified_Fungi	unclassified_Fungi	unclassified_Fungi	unclassified_Fungi

Supplementary Table 7 Overview of metagenomic data

Data processing steps	Overview of shotgun metagenomic data	Value
Quality control	Clean data base (bp)	89875897082
	No host clean data base (bp)	62,566,209,710
	Number of reads	418,568,454
Assembly	Contigs (≥300bp)	6106386
	Total length (≥300bp)	4,109,591,609
	GC (%)	49.85%
	N50	703
Annotations	Predicted genes (≥100bp, non-redundant)	5,377,218
	Assigned to NCBI nr database (%)	69.57%
	Assigned to KEGG database (%)	29.26%
	Assigned to CAZyme database (%)	11.23%

Supplementary Table 8 Top 20 high abundance (relative abundance > 0.1%) difference KOs and their functions

KO id	KO function	baseMean	Relative abundance	Enrichment
K00937	polyphosphate kinase	827.439	0.106	enriched
K01256	aminopeptidase N	1204.373	0.157	enriched
K02014	iron complex outermembrane recepter protein	8008.532	0.931497	enriched
K02033	peptide/nickel transport system permease protein	994.605	0.1275316	enriched
K02035	peptide/nickel transport system substrate-binding protein	1324.754	0.17	enriched
K02529	LacI family transcriptional regulator	1579.414	0.207	enriched
K03088	RNA polymerase sigma-70 factor, ECF subfamily	3216.83	0.423	enriched
K03294	basic amino acid/polyamine antiporter, APA family	953.672	0.127	enriched
K03466	DNA segregation ATPase FtsK/SpoIIIE, S-DNA-T family	842.403	0.109	enriched
K03585	membrane fusion protein, multidrug efflux system	850.753	0.112	enriched
K03832	periplasmic protein TonB	1226.814	0.164	enriched
K03924	MoxR-like ATPase	1026.459	0.133	enriched
K07058	membrane protein	914.818	0.119	enriched
K07090	uncharacterized protein	921.89	0.118	enriched
K07386	putative endopeptidase	1096.176	0.142	enriched
K15726	cobalt-zinc-cadmium resistance protein CzcA	902.059	0.121	enriched
K02256	cytochrome c oxidase subunit 1	3989.525	0.112	depleted
K03879	NADH-ubiquinone oxidoreductase chain 2	4701.959	0.107	depleted

Supplementary Table 9 Annotation of iron transport–related functional genes in bin0.

#Bin_ID	gene_ID	KO	KO_function	kegg_target_id	Percent-identity	Query-cover
C4_bin0	C4|protein000070928	K02014	iron complex outermembrane recepter protein	pagg:AL522_12275	96.72	100
C4_bin0	C4|protein000091963	K02014	iron complex outermembrane recepter protein	pva:Pvag_1276	94.92	94.15
C4_bin0	C4|protein000112449	K02014	iron complex outermembrane recepter protein	pagg:AL522_01255	92.97	100
C4_bin0	C4|protein000176950	K02014	iron complex outermembrane recepter protein	pva:Pvag_0188	94.59	100
C4_bin0	C4|protein000268039	K02014	iron complex outermembrane recepter protein	pva:Pvag_pPag30472	97.21	99.44
C4_bin0	C4|protein000373478	K02014	iron complex outermembrane recepter protein	pagg:AL522_01255	92.97	100
C4_bin0	C4|protein000393052	K02014	iron complex outermembrane recepter protein	pva:Pvag_0188	94.59	100

Supplementary Table 10 Results of linear mixed effects model for the main effects of P1, P2, B, and their two-way and three-way interactions on plant defense enzyme activities, plant hormone content and microbial community of common vetch. *P*-values < 0.05 are highlighted in bold.

Dependent variable	P1		P2		B		P1 × P2		P1 × B		P2 × B		P1 × P2× B	
	F	*P*	*F*	*P*	*F*	*P*	*F*	*P*	*F*	*P*	*F*	*P*	*F*	*P*
Incidence	10.863	**0.002**	4.959	**0.032**	10.863	**0.002**	4.198	**0.048**	19.051	**<0.001**	16.068	**<0.001**	0.071	0.791
Disease index	32.234	**<0.001**	1.077	0.306	49.289	**<0.001**	7.547	**0.009**	29.521	**<0.001**	35.067	**<0.001**	1.972	0.161
Abandunce of *C. lentis*	224.506	**<0.001**	315.97	**<0.001**	246.24	**<0.001**	232.928	**<0.001**	28.593	**<0.001**	213.868	**<0.001**	167.71	**<0.001**
Height	4.774	**0.035**	0.201	0.657	0.393	0.534	4.957	**0.032**	0.002	0.962	0.485	0.49	1.522	0.224
Fresh weight	0.37	0.547	1.293	**0.263**	44.055	**<0.001**	4.588	**0.039**	5.791	0.022	4.817	**0.035**	0.672	0.418
SOD	39.477	**<0.001**	125.226	**<0.001**	18.04	**<0.001**	20.961	**<0.001**	0.003	0.959	44.249	**<0.001**	1.201	0.28
POD	2.682	0.109	69.234	**<0.001**	13.087	**<0.001**	1.982	0.167	5.051	**0.03**	0.444	0.509	0.091	0.765
PPO	0.055	0.816	0.125	0.725	98.348	**<0.001**	14.674	**<0.001**	15.032	**<0.001**	344	0.562	34.316	**<0.001**
CAT	46.46	**<0.001**	0.262	0.612	16.114	**<0.001**	0.018	0.894	80.488	**<0.001**	21.724	**<0.001**	49.445	**<0.001**
JA	1.738	0.196	69.031	**<0.001**	13.239	**<0.001**	0.62	0.436	4.362	**0.044**	6.464	**0.016**	12.904	**<0.001**
SA	0.207	0.652	76.211	**<0.001**	30.93	**<0.001**	41.623	**<0.001**	17.054	**<0.001**	6.765	**0.014**	0.028	0.867
ET	5.088	**0.03**	0.005	0.945	64.086	**<0.001**	115.53	**<0.001**	12.226	**0.001**	30.872	**<0.001**	52.341	**<0.001**
CTK	2.044	0.162	6.065	**0.02**	108.937	**<0.001**	0.0445	0.834	30.528	**<0.001**	5.235	**0.028**	0.149	0.702
B_Chao1	9.432	**0.004**	0.796	0.378	3.58	0.066	6.049	**0.018**	0.811	0.373	0.099	0.754	1.726	0.196
B_Simpson	2.501	0.123	6.498	**0.015**	4.987	0.032	22.661	**<0.001**	120.17	**<0.001**	1.136	0.294	20.218	**<0.001**
B_positive cohesion	0.033	0.857	1.836	**0.183**	1.952	0.17	0.461	0.388	9.684	**0.003**	0.741	0.395	3.651	0.063

Supplementary Table 11 Primer sequences used in this study

Gene	Forward primer sequence	Reverse primer sequence
Epiphytic-16S (338F/806R)	ACTCCTACGGGAGGCAGCA	GGACTACHVGGGTWTCTAAT
Endophytic-16S (335F/769R)	CADACTCCTACGGGAGGC	ATCCTGTTTGMTMCCCVCRC
Epiphytic-ITS1 (ITS1F/ITS2)	CTTGGTCATTTAGAGGAAGTAA	GCTGCGTTCTTCATCGATGC
Endophytic-ITS1 (ITS1F/ITS2-Fungal-ad)	CTTGGTCATTTAGAGGAAGTAA	TGCGTTCTTCATCGATGC
ITS (ITS1/ITS4)	TCCGTAGGTGAACCTGCGG	TCCTCCGCTTATTGATATGC
16S (27F/1492R)	AGAGTTTGATCMTGGCTCAG	TACGGYTACCTTACGACTT
Q-*C. lentis*(CL1F/CL1R)	TGGTTGTTGTAGACGATGTGG	GGCAGAAGATTTACGACGACT

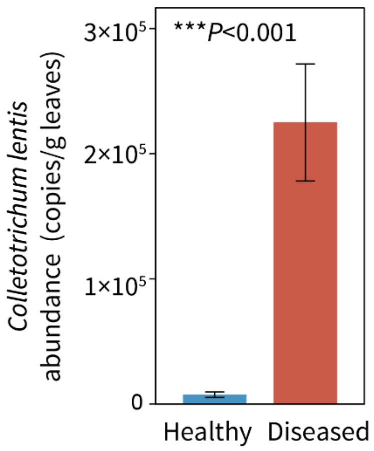

Supplementary Figure 1 The abundance of *Colletotrichum lentis* in healthy and diseased leaves of common vetch. *** above the bars means there was significant difference at *P*<0.001 using Student's t-test.

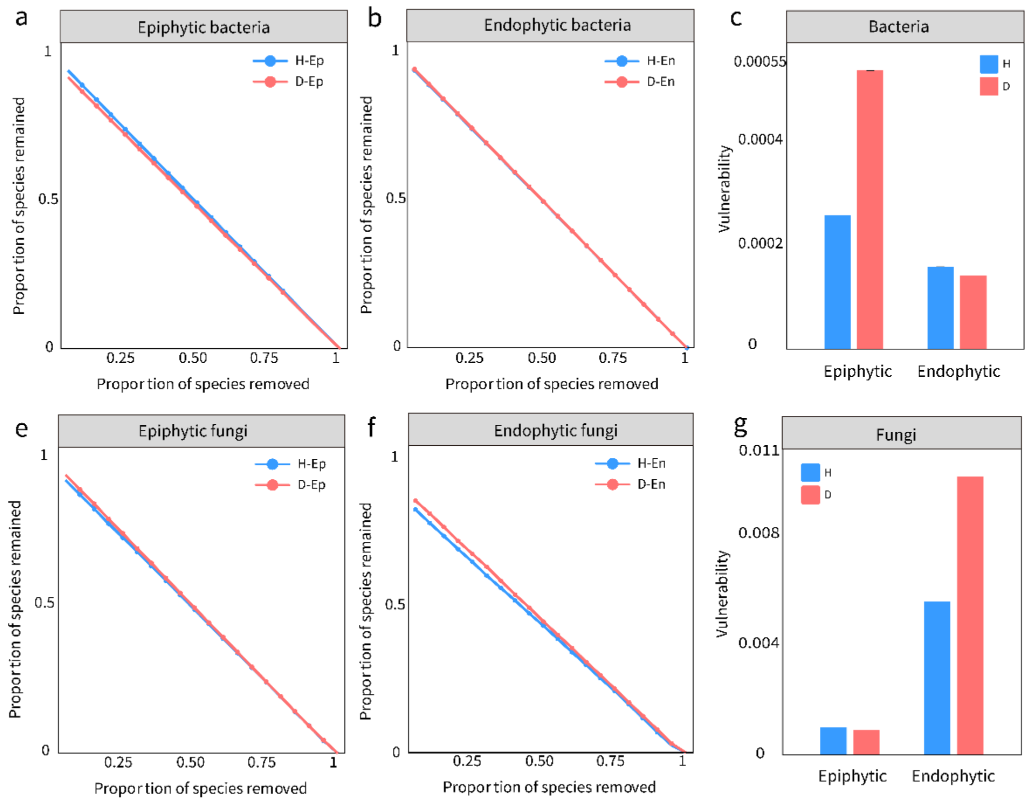

Supplementary Figure 2 Dynamics of network stability linking to network structure. Robustness of **a, b** bacterial and **d, e** fungal network measured as the proportion of taxa remained randomly removed from each of the network between healthy and diseased leaves. Extinction simulations were used to assess the robustness (resistance to node loss) of the microbial co-occurrence networks. **c** Bacterial and **f** fungal network vulnerability measured by maximum node vulnerability in each network.

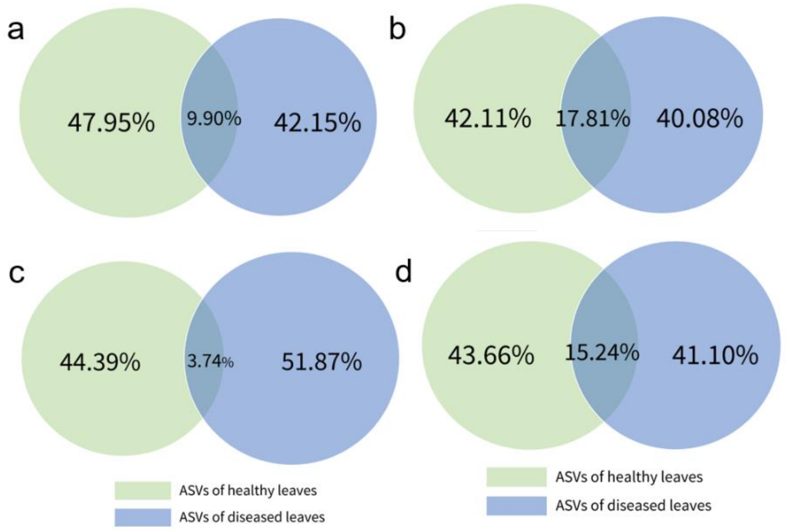

Supplementary Figure 3 Venn diagrams depicting the number of ASVs identified in **(a)** epiphytic bacteria, **(b) (c)**and **(d)** endophytic fungi of healthy and diseased leaves.

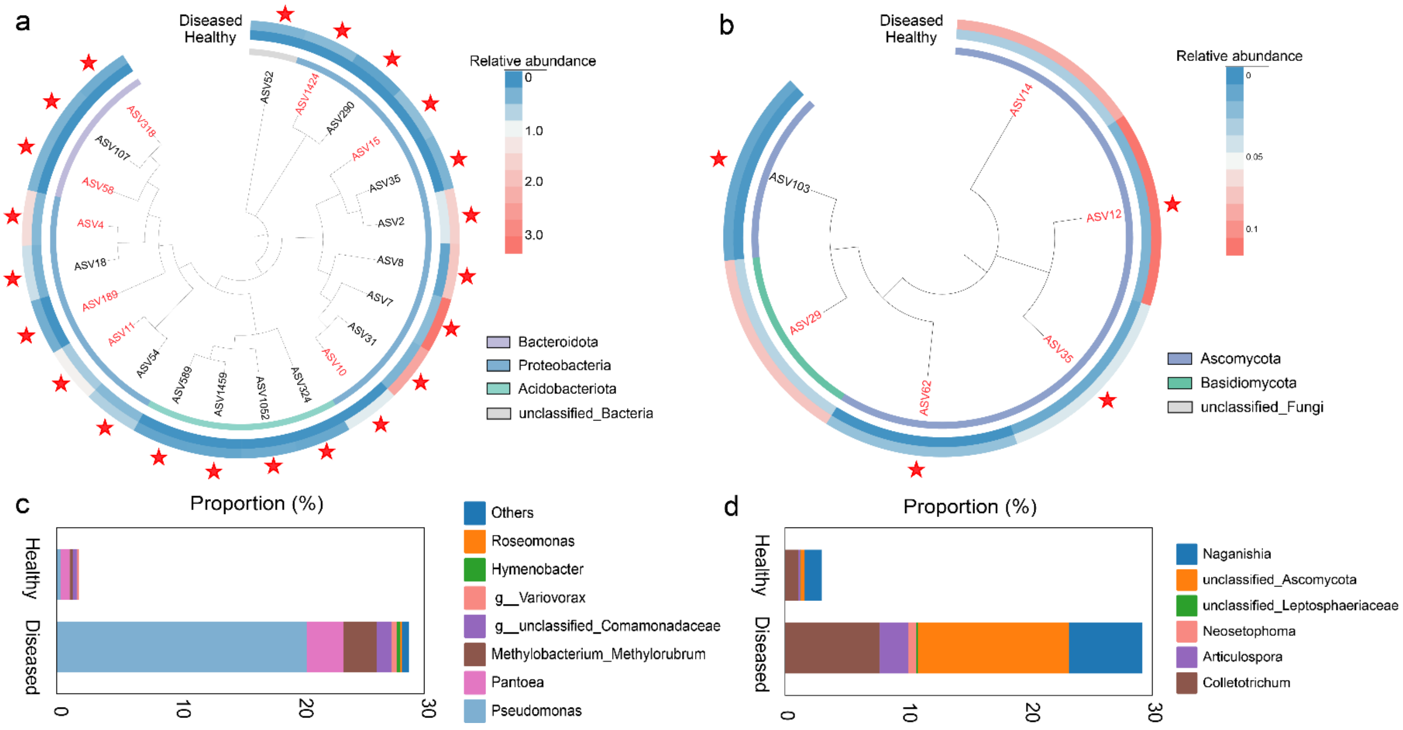

Supplementary Figure 4 Taxonomic characteristics of differential bacteria between healthy and diseased phyllosphere microbiotas. Identification of differential epiphytic bacteria ASVs (c) and endophytic fungal ASVs (d) between healthy and diseased leaves (relative abundance > 0.01%, sample prevalence > 1/2, |log2 fold change| ≥ 1, P < 0.05). ASVs highlighted in red indicate co-occurrence patterns with the pathogen, and red star denote ASVs that function as keystone hubs within the network. (e) Annotated genera of the enriched epiphytic bacterial ASVs and their proportion. (f) Annotated genera of the enriched endophytic fungal ASVs and their propotion.

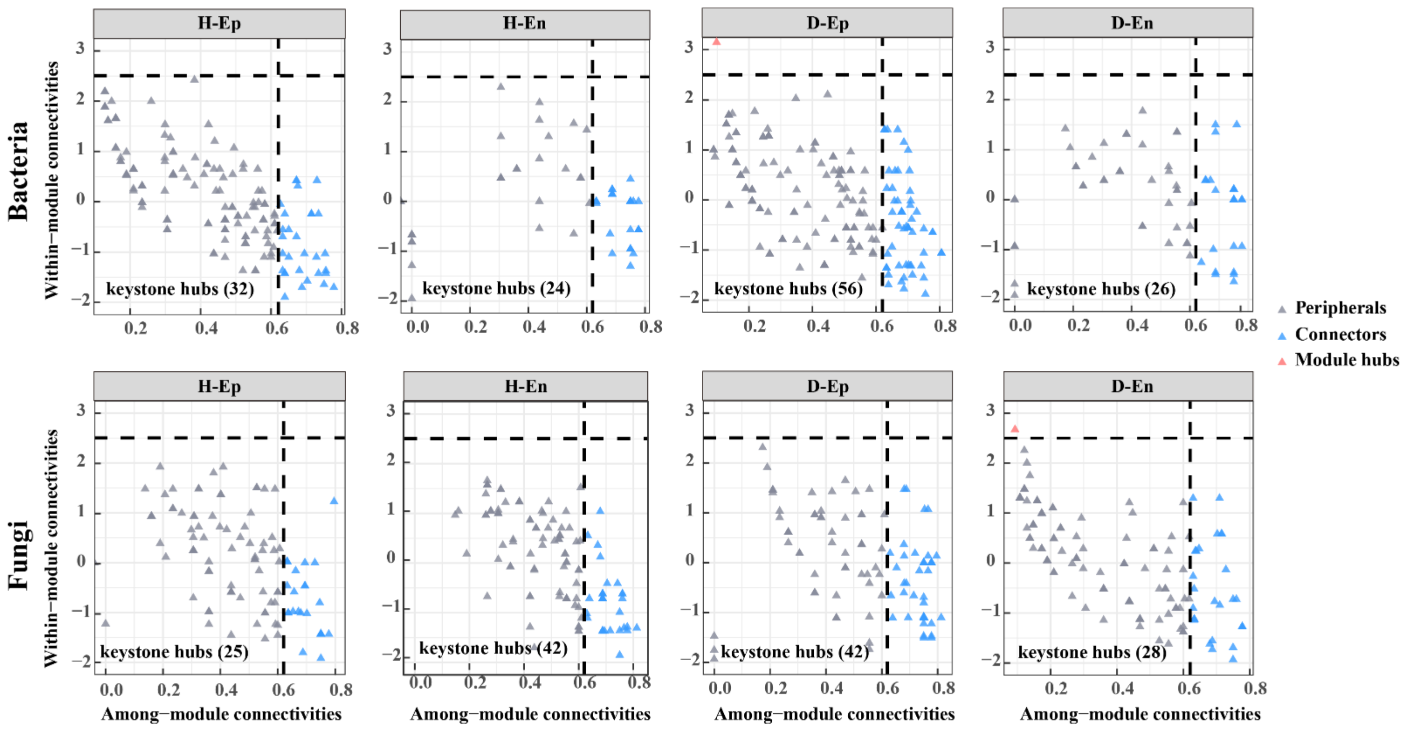

Supplementary Figure 5 The *Zi-Pi* method to reveal core hubs of bacterial and fungal network of common vetch (*Vicia sativa*) at different compartments between different healthy and diseased leaves.

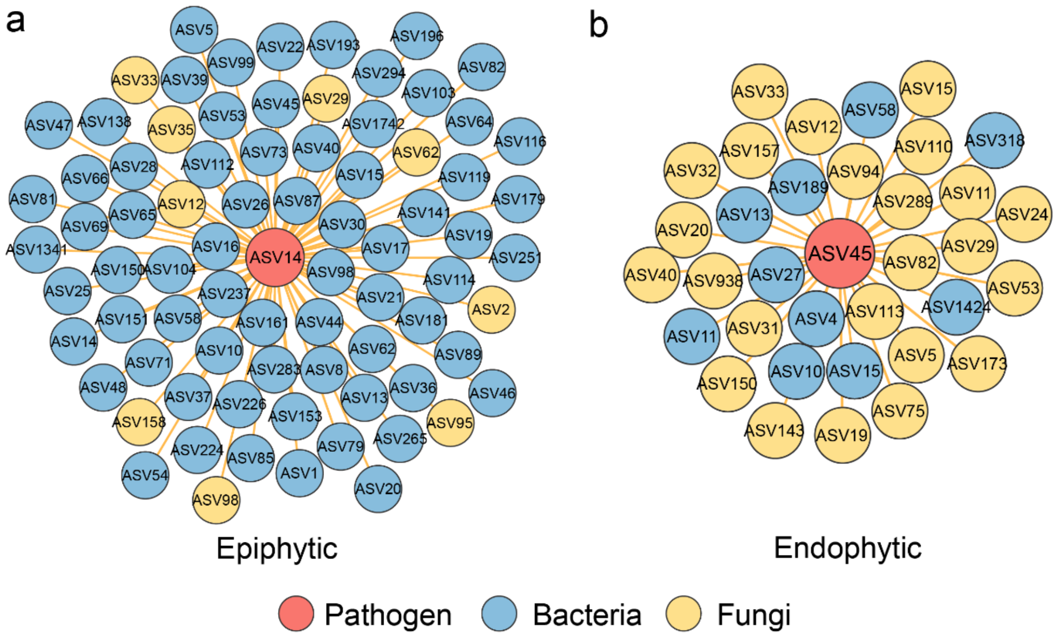

Supplementary Figure 6 Bacterial and fungal ASVs exhibiting co-occurrence patterns with the pathogenic ASV14 and ASV45 in the epiphytic and endophytic compartments under pathogen infection. Based on ITS sequence alignment, ASV14 and ASV45 were identified as the pathogenic taxa.

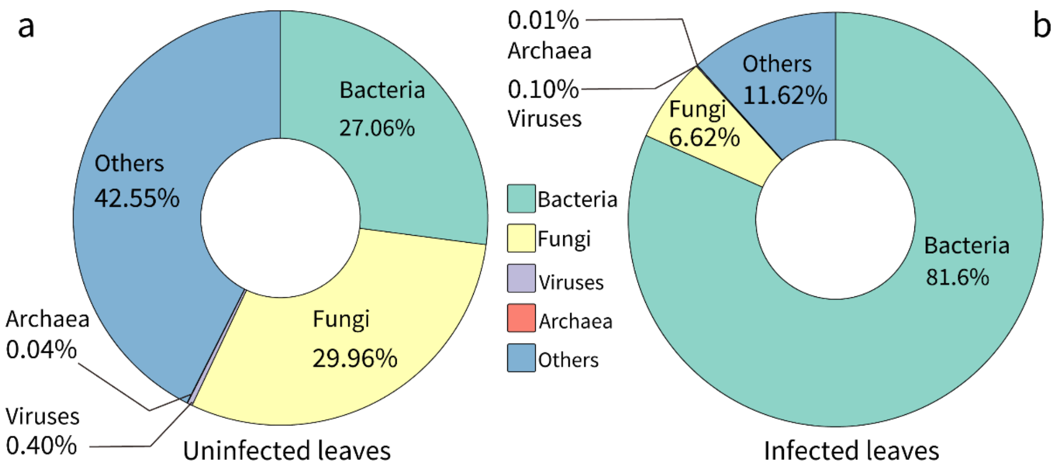

Supplementary Figure 7 Overview of the taxonomic classification in the epiphytic phyllosphere microbiome. Taxonomic classification was conducted by Kraken 2 against NCBI-nr database. The difference of taxonomic classification between **a** diseased leaves and **b** healthy leaves.

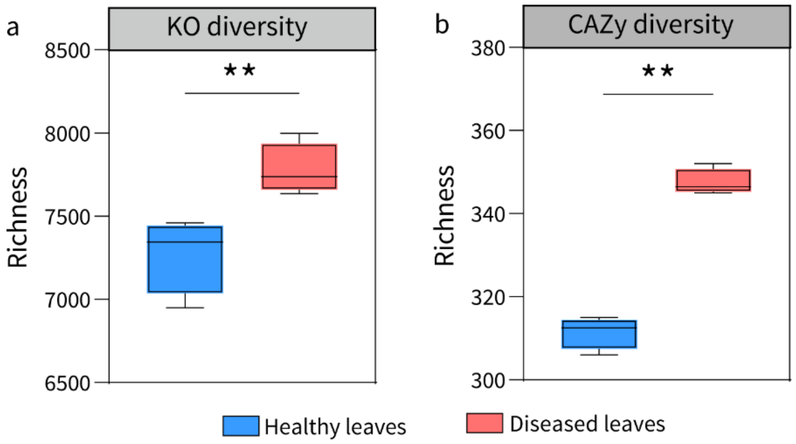

Supplementary Figure 8 Functional diversity in the epiphytic microbiome. Richness was conducted to characterize **a** KO and **b** CAZyme diversity and analyzed in Kruskal-Wallis to test for differences. Asterisks denote significant differences (**P* <0.05; ***P* <0.01; ****P*<0.001) and NS denotes no statistical significance.

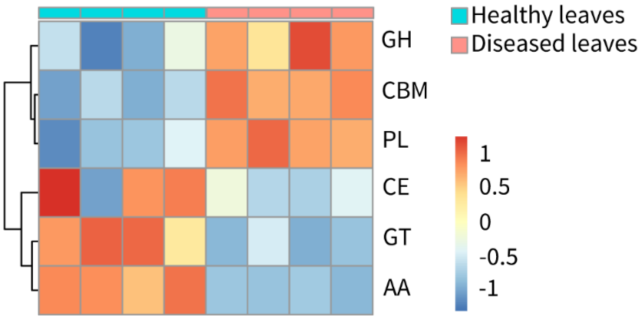

Supplementary Figure 9 The relative abundance of the glycoside hydrolases (GHs) and glycosyl transferases (GTs) functions in healthy and diseased leaves.

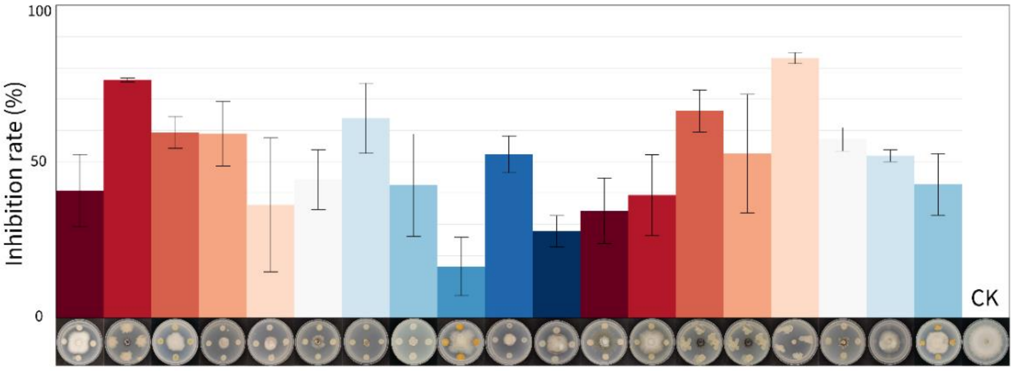

Supplementary Figure 10 The antagonistic activities in dual culture assays. A mycelial cake of *C. lentis* was placed in the center of the medium, and tested isolates were streaked on four ends. Plates inoculated with *C. lentis* only served as control.

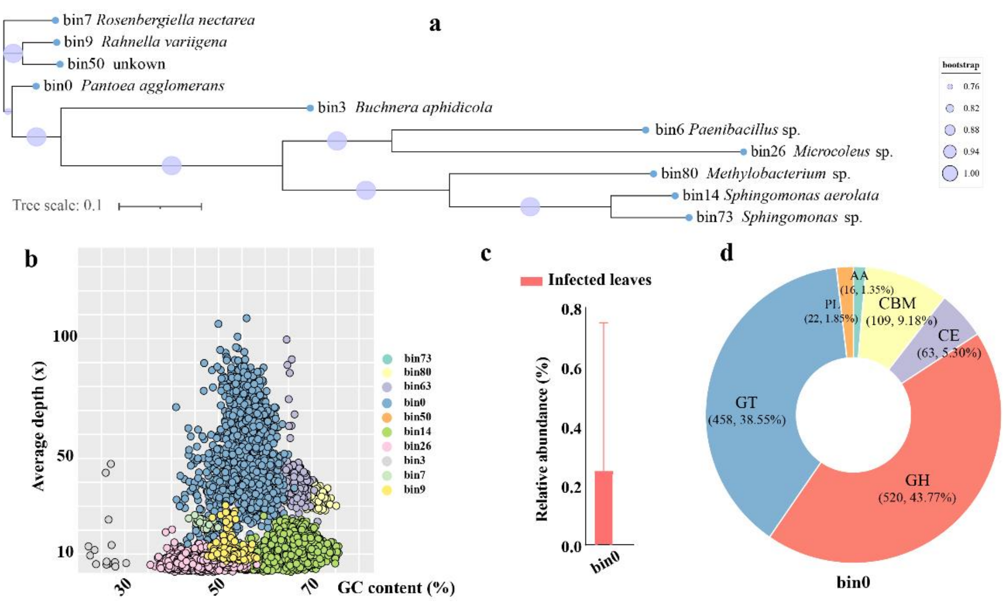

Supplementary Figure 11 Binning of the shotgun metagenomic data and reconstruction of the genomes. **(a)** Phylogenetic tree of the draft genomes (bins, n = 12) from the MAGs (metagenome assembled genomes) data (completeness > 80%, contamination < 10%). **(b)** Scatterplot representing the distribution of the assembled contigs based on their GC content and abundance. **(c)** The abundance (genome copies per million reads) of bin0 in the infected and uninfected samples. **(d)** Overview of Carbohydrate-Active Enzymes (CAZymes) functional profiling of the bacteria bin0.

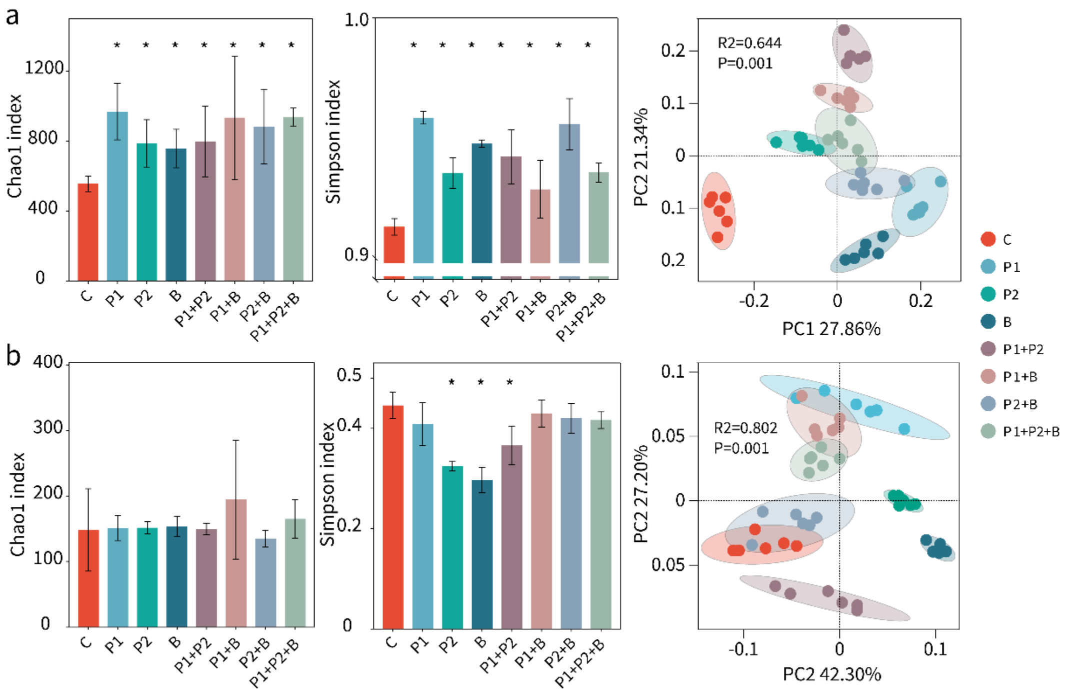

Supplementary Figure 12 The shift of microbial community after inoculating by different combination of SynComs.

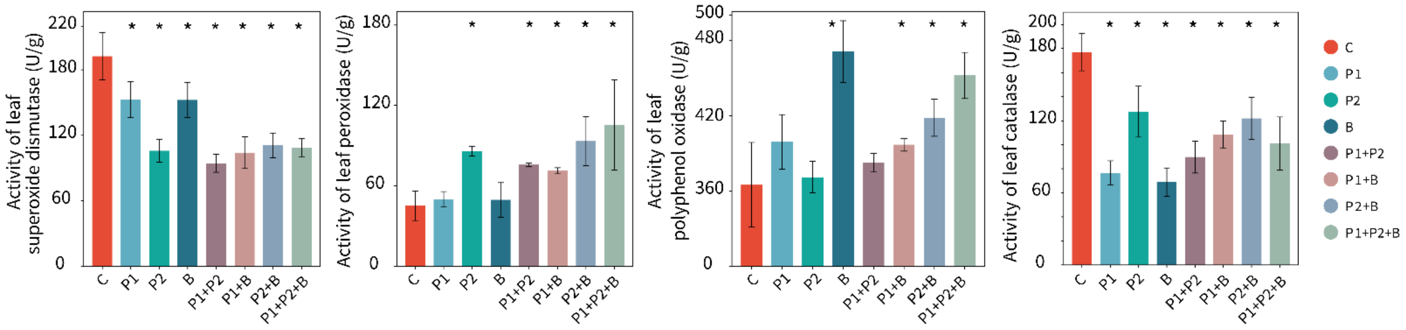

Supplementary Figure 13 The superoxide dismutase activity, peroxidase activity, polyphenol oxidase and catalase activity of common vetch after inoculating by different combination of SynComs. All the data were analyzed using Student's t-test. * means there was significant difference at $P<0.05$.

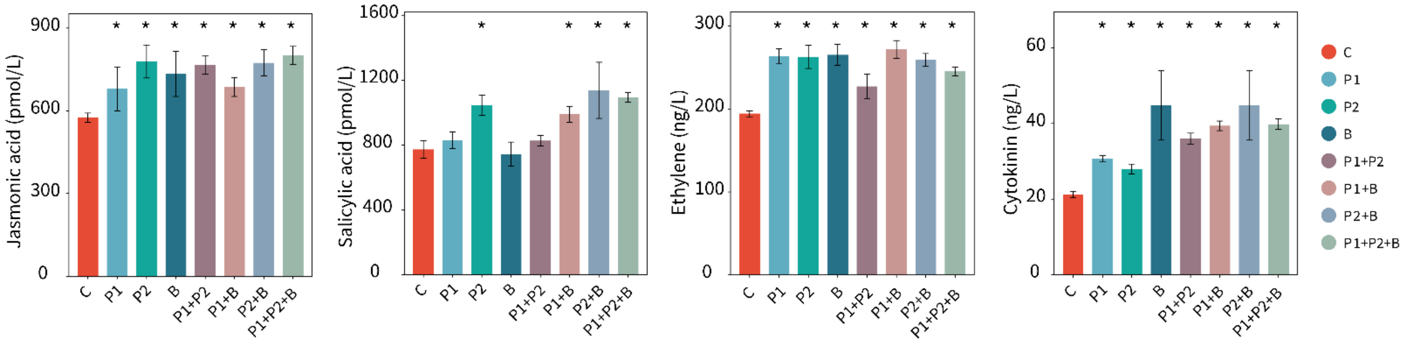

Supplementary Figure 14 The content of jasmonic acid, salicyline acid, ethylene and cytokinin of common vetch after inoculating by different combination of SynComs. All the data were analyzed using Student's t-test. * means there was significant difference at $P<0.05$.

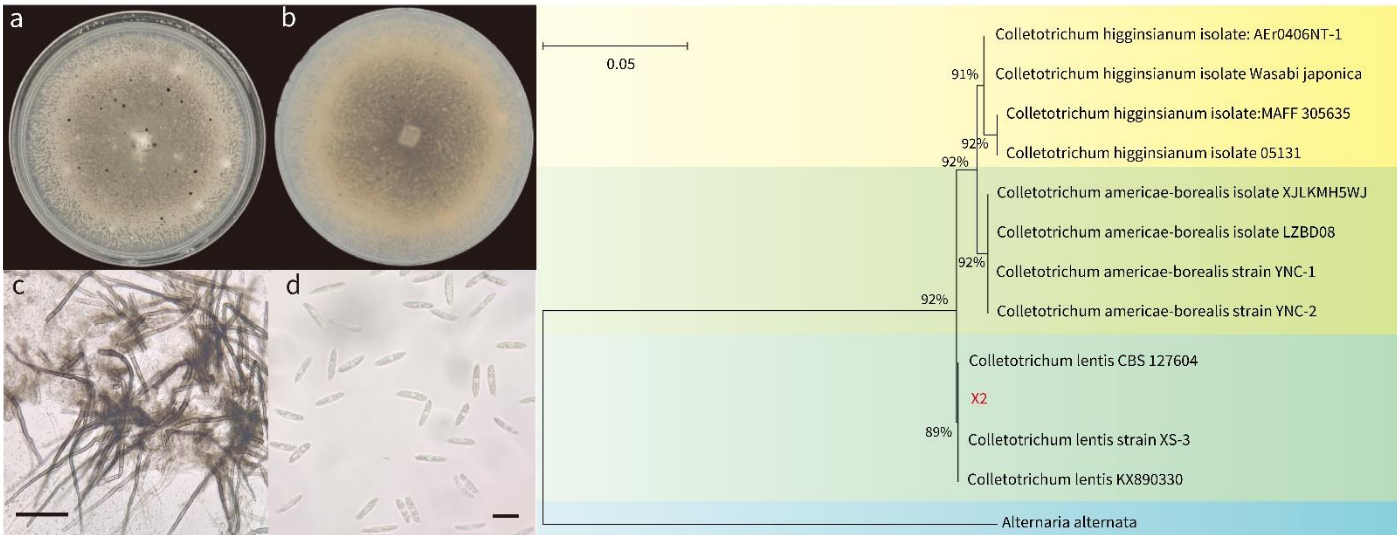

Supplementary Figure 15 Identification of *Colletotrichum lentis* (X2). Colony morphology on PDA (**a, b**; top and bottom view). **c** acervuli, **d** conidia. Scale bar of c = 100 μm, bar of d = 10 μm. **e** The maximum likelihood tree of *Colletotrichum* isolates.
